# Millimeter‐Scale Dual‐Opposing RNA‐Gradient Hydrogel for Interfacial Gene Silencing

**DOI:** 10.1002/advs.202524144

**Published:** 2026-04-07

**Authors:** Tyler Hoffman, Cong Truc Huynh, Marcus J. Goudie, Peyton J. Tebon, Hyojin Ko, Minh Khanh Nguyen, Kaelyn L. Gasvoda, Yang Song, Kirsten Fetah, Ali Khademhosseini, Song Li, Eben Alsberg

**Affiliations:** ^1^ Department of Bioengineering University of California, Los Angeles Los Angeles California USA; ^2^ Department of Biomedical Engineering University of Illinois at Chicago Chicago Illinois USA; ^3^ Jesse Brown Veterans Affairs Medical Center (JBVAMC) Chicago Illinois USA; ^4^ Department of Biomedical Engineering Case Western Reserve University Cleveland Ohio USA; ^5^ Department of Medicine University of California Los Angeles California USA; ^6^ Departments of Mechanical & Industrial Engineering, Orthopaedic Surgery, and Pharmacology and Regenerative Medicine University of Illinois at Chicago Chicago Illinois USA

**Keywords:** biofabrication, gene silencing, gradient hydrogels, patterning, RNAi, tissue engineering

## Abstract

Tissue interface restoration poses significant challenges in tissue engineering, particularly in areas requiring gradients of cellularity, biochemical composition, and mechanical properties essential for tissue‐specific functions. Recent advancements in microfluidic technology have enabled the creation of hydrogels with spatially defined gradients of biological molecules for engineering gradient tissues that mimic the natural heterogeneity of the native extracellular matrix. However, these gradients are typically outside relevant millimeter length scales for biological interfaces. To address these challenges, a branched microfluidic system capable of generating millimeter‐scale hydrogels with dual‐opposing gradients of RNAi molecules nanocomplexed with thiolated polyethyleneimine is demonstrated. These nanocomplexes are incorporated into photocrosslinkable poly(ethylene glycol)‐diacrylate (PEG‐DA) monomer solutions, which are then injected and mixed within a PDMS microfluidic chip featuring a branched channel network. The process results in stable, linear, 3‐mm dual‐opposing RNA gradients within the hydrogel, which is subsequently photocrosslinked. The generated hydrogels demonstrate precise spatial regulation of gene expression within encapsulated cells, as confirmed by fluorescence analysis. This platform holds significant potential for engineering complex tissue constructs and enabling the targeted delivery of RNAi molecules, influencing both encapsulated and endogenous cells. This advancement could play a crucial role in the regeneration of critical tissue interfaces such as tendon‐to‐bone and cartilage‐to‐bone.

## Graphical abstract

A branched microfluidic platform enables the fabrication of millimeter‐scale hydrogels with dual‐opposing RNA‐gradients for precise spatial control of cell gene expression. This device allows for the generation of multiple constructs at once, offering potential for future engineering of complex tissue interfaces with improved physiological relevance.

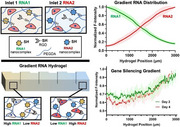

## 1 Introduction

Restoration of tissue interfaces after injury remains an ongoing challenge for the field of tissue engineering. Transitions between regions with distinct cellularity, biochemical components, and mechanical properties are first formed during development and are maintained over time [1]. Native gradients occur at these interfaces and are essential to support tissue‐specific function and longevity. Strategies to recapitulate the native tissue gradients have shown promise in improving the regeneration outcomes following interfacial tissue injuries [2].

Biofabrication techniques have been utilized to organize tissue‐specific biochemical and biophysical factors in a gradient fashion to better mimic endogenous tissue environments [3]. These have been achieved at a variety of scales, micron to centimeter, via control of diffusion under flow. Combinations of gradients within hydrogels, typically formed from synthetic or natural polymers, can be engineered to mimic native biochemical composition, tissue structure, and mechanical properties. Approaches to generate interfacial gradient hydrogels typically rely on pipette‐mediated input and subsequent diffusion through a bulk solution [4, 5]. This can be used to generate stacking approaches [6], where polymer gelation and diffusion have to be tightly controlled, often resulting in the presence of distinct regions with incremental changes in concentration. Discrete gradients are not representative of native tissue structure and can lead to phasic distribution of cell phenotypes, rather than a continuous transition. Additionally, it remains challenging to precisely control the dimensions of the gradient. Fluidic control, via syringe pumps [7, 8, 9], has been explored to control gradients. However, the dimensions of these hydrogels are often not suitable for the range needed for in vivo interfacial tissues, where gradient trends are generated in the 1–4 cm range [7, 8, 9, 10]. For example, the critical size defect for a rabbit osteochondral model is 3 mm, which may be challenging to fabricate using pre‐existing techniques.

RNA interference (RNAi) is a powerful tool permitting inhibition of gene expression at the post‐transcriptional level by the targeted degradation of specific mRNA molecules [11, 12, 13, 14, 15, 16, 17]. It has the potential to revolutionize the functional repair of damaged tissue by decreasing the expression of specific proteins that negatively impact healing processes or by altering stem cell differentiation pathways [11, 12, 13, 14, 15, 18, 19]. Hydrogels have been utilized to control the release of RNAi molecules, including short interfering RNAs (siRNAs) and microRNAs, to surrounding and encapsulated cells by mechanisms such as affinity or electrostatic interactions, diffusion through the biopolymer pores, and/or polymer degradation [11, 12, 13, 16, 17, 18, 19, 20, 21]. RNAi has been reported to guide human mesenchymal stem cells (hMSC) osteogenesis [12, 13, 14, 15, 22] and chondrogenesis [23, 24, 25] independently, and also be used for other applications, such as cancer treatments [26, 27]. For example, hydrogel‐mediated delivery of siRNA against Noggin (siNoggin) enhanced the osteogenic differentiation of encapsulated hMSCs [11, 12, 13, 14, 15, 22]. Recently, our groups have demonstrated the use of gradient distribution of siRNA against green fluorescent protein (siGFP) for controlling the corresponding gradient GFP expression of the encapsulated cells in the hydrogel constructs via a dual syringe pump approach [10]. Although the reported approach could demonstrate the feasibility of spatial regulation of cell gene expression via siRNA gradient presentation, this technique could not be scaled down to fabricate some gradient implants of physiologically relevant size. Unlike previous protein‐ or small molecule‐based gradients, our approach generates stable, millimeter‐scale dual‐opposing RNA gradients that provide post‐transcriptional regulation of cell behavior and sustained spatial control over gene silencing, addressing a key gap in tissue interface engineering. We introduce a strategy to covalently tether nanocomplexes of RNA and thiolated polyethyleneimine (RNA/PEI‐SH) into the hydrogel backbone, extending gradient stability and enabling sustained, spatially regulated gene silencing.

**FIGURE 1:**
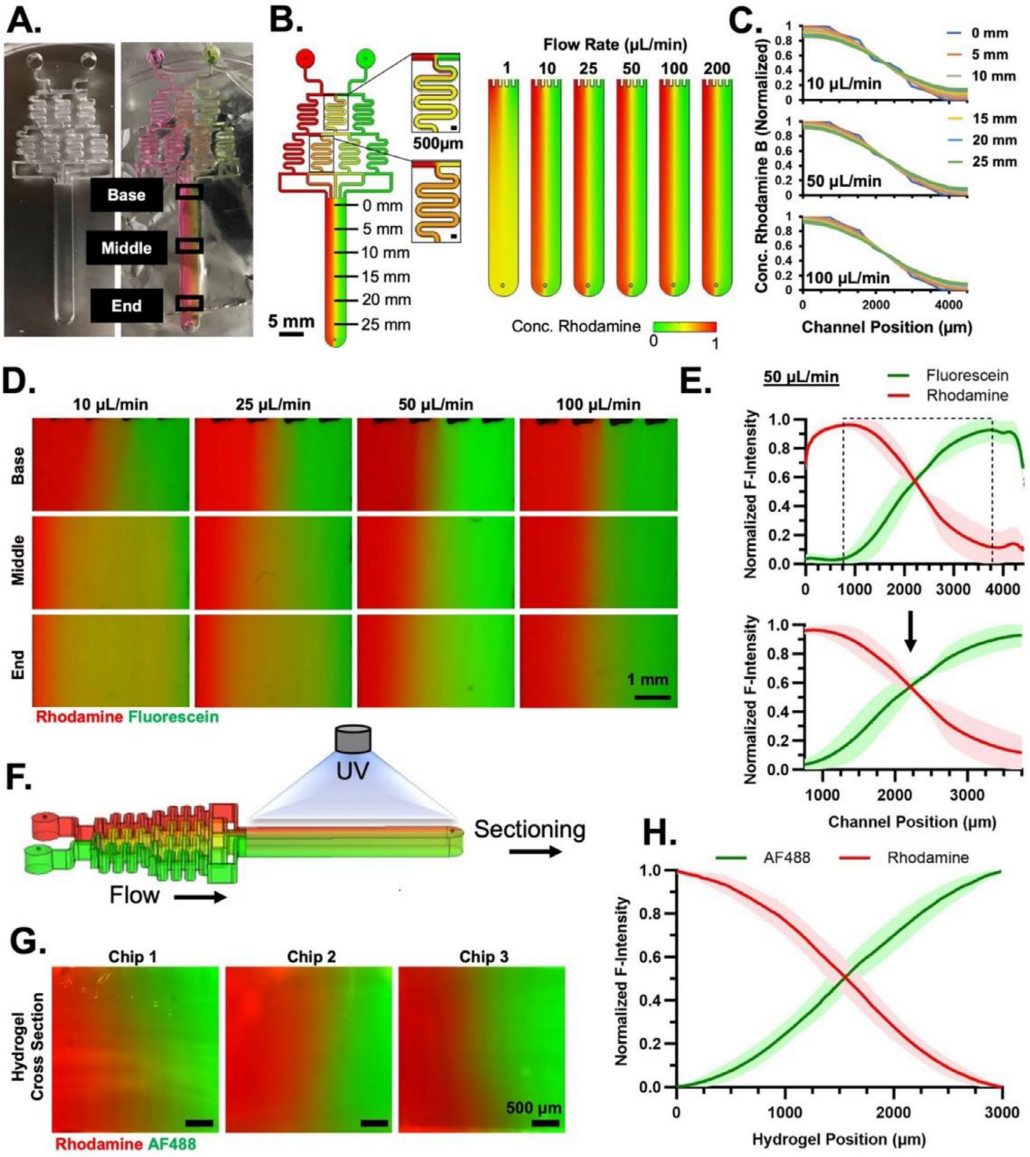
Fluidic Gradient Generation and Crosslinking. (A) PDMS fluidic mold after fabrication (left) and during a fluidic experiment with fluorophores (right). Black boxes indicate regions of interest where different gradient profiles were evaluated. (B) Design of the fluidic chip in COMSOL visualizing the different distributions of rhodamine concentrations within the crosslinking chamber at varying flow rates. (C) Quantified rhodamine concentration profiles across the width (4.5 mm) of the crosslinking chamber at varying positions generated via the COMSOL model. (D) Fluorescence microscope images taken to visualize the distribution of fluorophores rhodamine and fluorescein at varying positions and flow rates. (E) Distribution of fluorophore intensity across the entire width of the chamber (top) was quantified using the ImageJ line profile function. Plot represents average intensity with standard deviation based on *n* = 3 fluidic devices at 50 µL/min. The central 3 mm gradient profiles are highlighted (bottom). (F) Schematic of the crosslinking process, where first a gradient is generated in the chamber and crosslinked immediately following flow stoppage. Hydrogels are removed from the device and cross‐sectioned to generate individual hydrogels. (G) Fluorescence microscope images of representative cross‐sectioned hydrogels generated from three individual fluidic devices. (H) Distribution of fluorophore intensity across the gradient width of cross‐sectioned hydrogels quantified using the ImageJ line profile function. The plot represents the average intensity and standard deviation of 30 individual hydrogels from *n* = 3 fluidic devices.

In this study, we have developed a branched microfluidic system that can be used to generate hydrogels with dual‐opposing gradients of siRNAs at a clinically relevant size (Figure 1) that may be helpful in engineering complex tissue constructs. The PDMS microfluidic chips, fabricated by utilizing laser‐cutting in lieu of microfabrication techniques, have demonstrated their capability in generating millimeter‐scale hydrogels containing dual‐opposing gradients of two different siRNAs for spatially regulating cell gene expression at a physiologically relevant millimeter scale. The microfluidic chip involves the pump‐controlled injection of two siRNA‐laden photocrosslinkable PEG‐DA monomer solutions into a branched gradient device that consists of a network of channels that repeatedly split and mix the injected solutions [28]. After passing through the channels, the monomer solutions enter a larger crosslinking chamber, where a solution of stable dual‐opposing siRNA gradients is formed, followed by photocrosslinking to generate a hydrogel with designated siRNA gradients. The dual‐gradient of siRNAs within hydrogels is maintained over time. This work highlights the synergies between biochemical spatial presentation as well as UV light‐induced chemistries to crosslink the hydrogel and retain the input spatial presentation of biochemical factors in the intended geometries. This approach allows for the generation of hydrogel constructs with consistent linear 3 mm dual‐opposing gradients of two RNAi molecules in a high‐throughput fashion. The resulting gradient patterns were characterized with image analysis to verify the generation of robust continuous gradients. The spatial presentation of siRNA gradient in the fabricated hydrogels silenced the expression of fluorescence reporter gene in encapsulated cells in a corresponding spatial gradient. This approach allows the generation of dual‐opposing linear gradients of two different RNAi and/or biochemical molecules, which may be beneficial in engineering complex tissue constructs with continuous gradients from one tissue type to another. This would benefit interfaces such as tendon‐to‐bone, muscle‐to‐tendon, or cartilage‐to‐bone that transition in cellularity, composition, organization, and mechanical properties on the millimeter scale [29, 30]. This platform can be utilized to prepare gradients of RNAi molecules that influence encapsulated cells, and it is anticipated that this technology may be used to influence endogenous cells following implantation for tissue regeneration.

## 2 Results and Discussion

### 2.1 Modeling of Microfluidic Chip

To fabricate a millimeter‐scale gradient hydrogel, we first modeled a microfluidic chip mixer that aligned with our design criteria. A microfluidic chip device (Figure 1A) was designed, which can be fabricated from PDMS using laser‐cut acrylic molds, which could convert two input polymer solutions with soluble siRNAs or biomolecules to a single hydrogel with continuous dual‐opposing gradients at a scale of 3 mm. At this scale, the engineered gradient hydrogels might be used for engineering small‐scale complex tissues or for in vivo spatial delivery of RNA gradients. The designed microfluidic device can discretely mix two input siRNA‐laden, photocrosslinkable macromer solutions (e.g., PEG‐DA) to achieve a 3 mm‐scale gradient across a crosslinking chamber of up to 30 mm in length. The channel network with a cross‐sectional area of 0.5 mm width × 3 mm height was kept constant throughout the device to maintain laminar flow throughout the chip at a wide range of flow rates. After mixing through sequential mixing‐dividing channels, five final channels feed into the crosslinking chamber for a final cross‐sectional area of 4.5 × 3 mm. In this crosslinking chamber, the solution of dual‐opposing siRNA gradients was generated and photocrosslinked to fabricate a dual‐opposing siRNA‐gradient hydrogel. Based on this design, laminar flow was confirmed throughout the device at flow rates from 1 to 200µL/min (Re<1 at the highest flow speed).

We employed COMSOL to model the diffusion of solutes within the microfluidic chip under flow conditions (Figure 1B,C). Complete mixing occurred within the branching structures at a wide range of flow rates from 1 to 200 µL/min. In each flow rate case, five distinct concentrations are achieved in the five channels feeding into the crosslinking chamber. However, significant differences in the gradient formation and maintenance from the proximal and distal ends of the crosslinking chamber were observed, based on the flow rate and size of the molecule. The percent change in the gradient slope from the 0 mm position to the 25 mm position was calculated at different flow rates, modeling rhodamine B (∼0.5 kDa) as a representative small molecule and siRNA/PEI nanocomplexes (250 nm diameter) as the molecule we intend to use in our application. With higher flow rates and larger molecule sizes (Figure S1A–C), discrete concentrations are more obvious near the proximal end at the 0 mm position but become mixed to form maintained continuous gradients by the 25‐mm position. The percent change between the 0‐ and 25‐mm positions was minimized for both rhodamine B and siRNA/PEI nanocomplexes at a 50 µL/min flow rate (Figure S1D). Modeling of PEI (MW 25 kDa), chosen for its size similar to growth factors and cytokines, was also conducted, and the relative changes from the 0‐ to 25‐mm positions are minimized at 50 µL/min. This led to a 20% reduction in gradient slope from the proximal to distal end, or a 10% change in maximum concentration at the highest and lowest points of the 4.5 mm gradient between proximal and distal chamber ends. This model also confirmed a lack of discrete concentration changes, even for the largest 250 nm molecule size, which is important for modeling biological interfaces such as the osteochondral tissue, which involves a smooth transition from bone to cartilage tissues. At the 50 µL/min flow rate, sufficient time for diffusion is provided, enabling the five initial concentrations to mix and form a continuous gradient throughout the chamber.

### 2.2 Experimental Fluidic Optimization

We proceeded with the fabrication of this microfluidic device based on the specifications confirmed via our COMSOL model. Briefly, an acrylic template (3.2 mm in thickness) was laser cut to generate our intended design with 0.5 × 3.0 mm channels, and a 4.5 × 3.0 mm crosslinking chamber with 30 mm in length. Laser‐cut molds were bonded to an acrylic base (40 × 80 mm) using acrylic cement to fabricate the negative of the PDMS fluidic device (Figure 1A). The use of laser‐cut molds allows for the generation of a millimeter‐scale fluidic mixer, which exceeds the size limitations of standard photolithography techniques. This scale of the mold is important for achieving hydrogels with dimensions relevant to in vivo interfacial tissue models, such as the size of osteochondral defects in small animals. This study establishes the proof‐of‐concept for fabricating dual‐opposing RNA gradient hydrogels capable of silencing cell gene expression at millimeter scales. To validate our device, rhodamine B (Red, 479 Da) and green fluorescein (Green, 332 Da) were utilized as representative small molecule indicators for testing the formation of gradient signals in the crosslinking chamber and optimizing the fabrication process in the microchips with photocrosslinkable PEG‐DA as a macromer. The flow rate of macromer solutions through the chip was varied in a range of 10–100 µL/min, and the distribution of fluorescent signal was evaluated via fluorescence imaging. Red‐ and Green‐indicator PEG‐DA solutions (indicator 0.01 mg/mL, PEG‐DA 10% w/v) served as the two inlet solutions, which were injected into the microchip inlets via syringe pumps. Figure 1A shows the PDMS chips before and after filling with macromer solutions containing 2 indicators with opposite gradient distributions. The fluorescent signals were recorded from three locations (the base, middle, and end) in the chips and normalized to the maximum signal of each fluorophore across all positions to observe fluorescent distribution. The fluorescence signals from the three locations demonstrated the formation of dual‐opposing gradients of two indicators along the length of the whole crosslinking chamber (Figure 1D,E). The flow rates of macromer inlet solutions significantly impact the slope of the formed gradients along the crosslinking chamber from the proximal (base) to distal (end) positions, demonstrated via quantified relative fluorescence signals in the obtained images spanning the entire 4.5 mm crosslinking chamber (Figure S2A). With lower flow rates of 10 and 25 µL/min, significant differences between the base and end locations were observed, where significant overmixing was observed near the end of the chamber. At 50 and 100 µL/min, continuous near‐linear dual gradients were observed with minimal changes at the different positions. The continuous gradients established using a 50 µL/min flow rate were achieved in three separate devices with a minimal difference (Figure 1E), demonstrating the reproducibility of the fabricated device. When the gradient profile was measured at 5 different positions in each device along the crosslinking chamber (Figure S2B), the establishment of dual‐opposing gradients of small molecules across the entire crosslinking chamber was achieved with a maximum variability of ∼10% while using a flow rate of 50 µL/min. Additionally, the near‐linear dual‐gradient profiles were observed when focusing on the central 3 mm of the whole 4.5 mm gradient across the chamber, compared to the sigmoidal curves observed in the outer 750 µm in both fluorophore profiles. The gradient trends of rhodamine and fluorescein fit a linear trendline with an R^2^ of 0.857 and 0.861, respectively (Figure S2C). Moving forward, we focused on the gradient profile within the central 3 mm to best optimize the linear gradient profile.

### 2.3 Crosslinking of Small‐Molecule Gradient Hydrogel

We next wanted to demonstrate that the dual gradient could be retained within a hydrogel following crosslinking and harvesting of the gel from the fluidic device. Initial screening identified that fluorescein was sensitive to photobleaching following UV exposure, so we proceeded with AlexaFluor 488 (AF488, green) as a representative small molecule fluorophore. The fluidic chip was primed with rhodamine and AF488 dye in PEG‐DA (final 10%, w/v, supplemented with 1 mg/mL of lithium phenyl (2,4,6‐trimethylbenzoyl) phosphinate (LAP) as a photoinitiator for UV crosslinking) solutions at 50 µL/min. Following the establishment of a gradient, the crosslinking chamber was exposed to 110 mW/cm^2^ UV light for 20s to induce gelation (Figure 1F). Rapid crosslinking is required to prevent excess diffusion, while lower UV intensities were not sufficient for crosslinking and maintaining the gradient after crosslinking. The bulk hydrogel (3.0 × 4.5 mm with 30 mm length) was removed from the fluidic device and imaged with a fluorescence microscope at different positions to visualize the distribution of each fluorophore. Images of the whole bulk hydrogel taken at positions 1–5 were analyzed via ImageJ to determine the relative intensity of signal across the central 3 mm of the hydrogel (Figure S3A). We did not observe significant changes in gradient distribution across the different positions along the crosslinking chamber, indicating a consistent amount of small molecule concentration of both fluorophores from the base to the end of the hydrogel. Next, the harvested hydrogels were sectioned into individual 3 mm length pieces by cutting parallel to the gradient axis. In this way, the cross‐section of each hydrogel would contain a dual gradient of both molecules and could be evaluated independently. Ten gels from each of three different chips were generated and imaged via fluorescence microscope to observe the gradient profile. Representative images from one cross‐sectioned hydrogel from each of three devices are shown in Figure 1G. We focused on the central 3 mm region to best evaluate the linearity of the dual‐gradient profiles as well as exclude observed fluorescence edge effects. Here, fluorescence intensities were normalized within each individual gel to visualize the consistency of dual‐gradient profiles. Figure 1H demonstrates that both of the gradients demonstrated near‐linear continuous profiles with <8% standard deviation across multiple chips. The gradient trends of rhodamine and AF488 fit to linear trendlines with R^2^ of 0.9632 and 0.9674, respectively (Figure S3B). The difference in fluorescence slopes between both fluorophores was not statistically significant, indicating both inlets lead to uniform gradients of each molecule (Figure S3C). These results indicated that our developed approach could fabricate multiple hydrogels at once with dual‐opposing gradients of two molecules in a consistent fashion.

### 2.4 Crosslinking of siRNA/PEI‐SH Nanocomplexes Gradient Hydrogel

**FIGURE 2:**
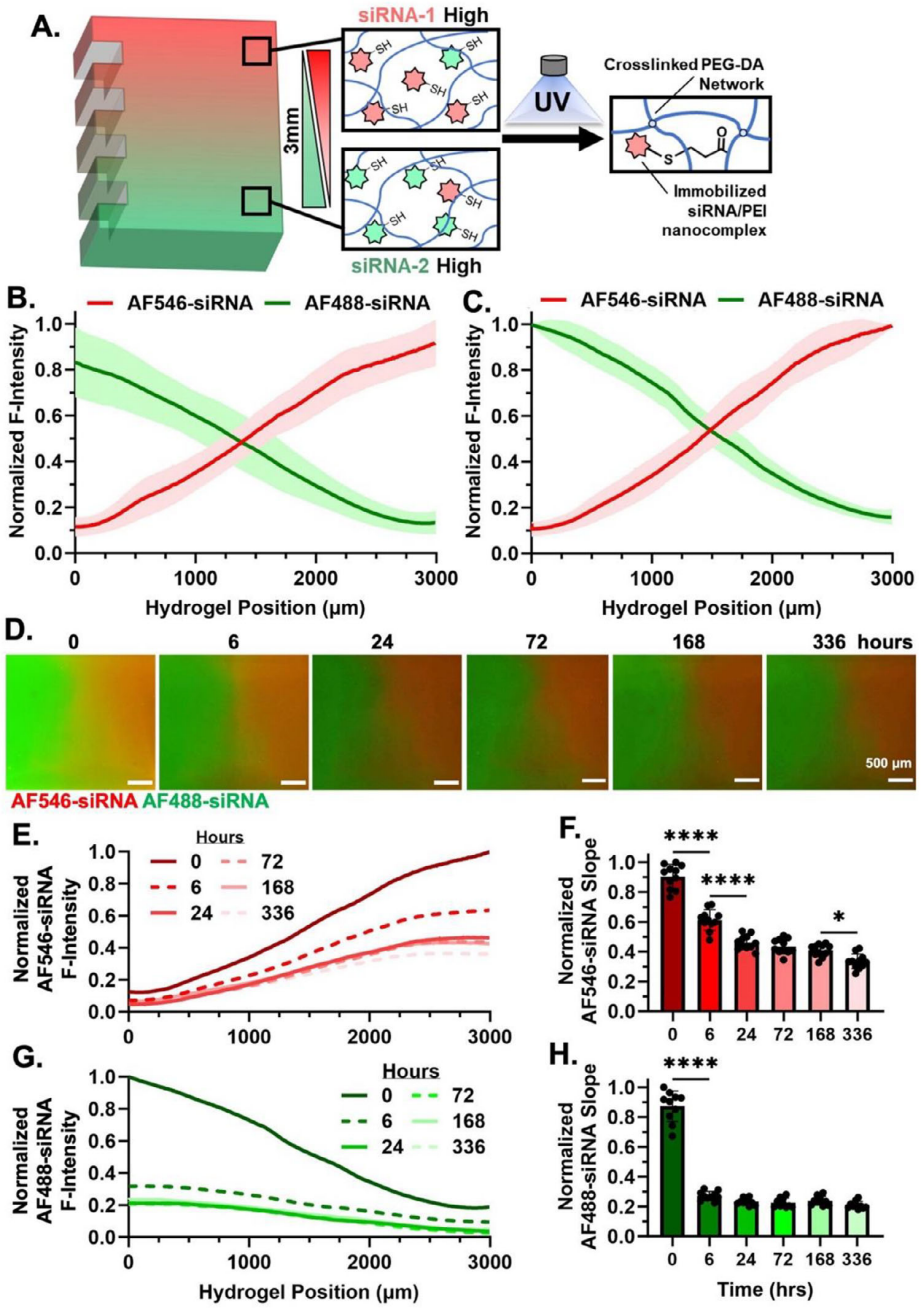
Fabrication of Dual‐opposing siRNA‐Gradient Hydrogel. (A) Schematic of a dual‐opposing siRNA‐gradient hydrogel during the fabrication process. The thioether linkage formed from the thiol group in PEI‐SH and the acrylate group in PEG‐DA during UV crosslinking of PEG‐DA hydrogels helps to immobilize and reduce the diffusion of tethered siRNA/PEI nanocomplexes from the hydrogel matrix and maintain the dual‐opposing siRNA gradients. Distribution of normalized fluorescent signal from siRNA across the gradient width of the hydrogel observed from the (B) bulk view and (C) cross‐section view, quantified using ImageJ. Plot represents average normalized intensity and standard deviation of (B) positions from each of 3 individually fabricated fluidic hydrogels (*n* = 13 positions, normalized to global maximum) or (C) individual sectioned hydrogels generated from 3 individually fabricated fluidic hydrogels (*n* = 10 sections, normalized to maximum within individual sections) immediately following fabrication. (D) Fluorescence microscope images of representative cross‐sectioned hydrogels showing the retention of siRNAs at varying time points following fabrication (0 h) and incubation in PBS (pH 7.4) at 37°C (6–336 h). Distribution of (E) AF546‐siRNA and (G) AF488‐siRNA normalized intensity across the gradient width of the hydrogel over time. The plot represents an average normalized intensity profile of 10 individual sectioned hydrogels generated from 3 individual fluidic hydrogels, normalized to the maximum signal of each gel at each time point. Slopes of (F) AF546‐siRNA (*n* = 11) and (H) AF488‐siRNA (*n* = 10) fluorescence intensity profiles at varying time points (^*^ *p* < 0.05, ^****^ *p* < 0.0001).

Our next goal was to demonstrate the generation and retention of dual‐opposing gradients of siRNA/PEI nanocomplexes within a photocrosslinked PEG‐DA chip hydrogel. AF488‐ (Green) and AlexaFluor 546 (AF546, Red)‐tagged siRNAs were used as indicators to allow the use of fluorescent images to determine distribution in the hydrogels. AF546‐tagged siRNA was utilized in lieu of rhodamine‐tagged siRNA due to its commercial availability. PEI has been widely used as a transfection reagent for RNA delivery [12, 13, 14, 15, 31, 32, 33]. We have previously demonstrated that the presentation of RNAi molecules in the hydrogel was significantly prolonged by covalently tethering RNA [16] or affinity molecules [17, 33] into the backbone of the network. To prolong the presentation of gradient siRNA distribution in the hydrogel construct, we have developed PEI‐SH that could not only be used as an siRNA transfection reagent but also as a tethering molecule to provide a covalent linkage with the hydrogel network via thiol‐acrylate reaction during the crosslinking step to minimize RNA diffusion (Figure 2A). siRNAs were nanocomplexed with PEI‐SH at an N:P ratio of 11, as was used in our previous reports [12, 13, 14, 15]. Solutions of PEG‐DA (final 10% w/v) containing siRNA/PEI‐SH nanocomplexes (40 µg/mL or 3.2 µm) were flowed into the microfluidic chip at 50 µL/min, followed by crosslinking using the previously optimized parameters for rapid gelation. Again, fluorescence was utilized to easily visualize the distribution of siRNA molecules within the hydrogel. These fluorescently labeled siRNAs are photostable after UV crosslinking and maintain fluorescent intensity in culture for at least 18 h (Figure S4A,B). The bulk hydrogels and sectioned hydrogels were imaged and analyzed similarly to the small molecule fluorophore experiments. In the bulk view, a global maximum was used to normalize the intensities for measuring the relative fluorescence intensity along the length of the crosslinking chamber. A consistent linear gradient across different fabricated fluidic hydrogels was observed, with maximum concentration variance of 14.9% for AF488‐siRNA (6 µg/mL variance) and 10.1% for AF546‐siRNA (4 µg/mL variance) across the entire 30 mm hydrogel length (Figure 2B). However, the slope of the gradient was smaller (relative intensities span/range ∼0.8–0.1) compared to that of the small molecule (∼1.0–0). This is possibly due to the larger molecule size with less diffusion compared to that of rhodamine B and fluorescein. Characterization of hydrogels from cross‐section view further confirmed the presence of the dual‐opposing siRNA gradients in the fabricated hydrogels, and the slope was well maintained across different sections along the crosslinking chamber from three distinct fluidic hydrogels (Figure 2C). To confirm the prolonged maintenance of fabricated dual‐opposing siRNA gradients over time, the hydrogel sections were individually incubated in PBS at 37°C in wells of 24‐well plates and imaged sequentially at designated time points (Figure 2D). Quantification of the fluorescence signal of AF546‐siRNA shows that a continuous gradient was retained up to two weeks following fabrication, and the maximum intensity of the gradient as well as the slope of the gradient decreased over time (Figure 2E,F). Importantly, the continuous near‐linear gradient RNA distribution exhibited a decreasing slope over time, indicating release of siRNA from the hydrogel matrix contributed by the degradation of ester bonds formed between the RNA/PEI‐SH nanocomplexes and the hydrogel backbone. The largest statistically significant changes in gradient slope occurred between 0–6, 6–24, and 16–336 h, indicating the greatest change in siRNA release. The fluorescent signal of AF488‐siRNA demonstrated a gradient initially that was quickly lost within 6 h (Figure 2G,H). However, this loss of fluorescent signal is unlikely to be a result of AF488‐siRNA instability, as both AF488‐ and AF546‐siRNA demonstrated comparable stability (Figure S4B). Instead, the decrease is likely due to interference from PEG hydrogel autofluorescence in the 488 nm channel, which can confound fluorescence‐based visualization and may not accurately represent siRNA distribution, whereas AF546 provides a more reliable readout. These promising results indicate the potential to use this platform for generating dual‐opposing RNA‐gradient delivering hydrogels to knockdown gene expression of encapsulated and surrounding cells in gradient fashions.

### 2.5 Functionality of siRNA‐Gradient Hydrogel

**FIGURE 3:**
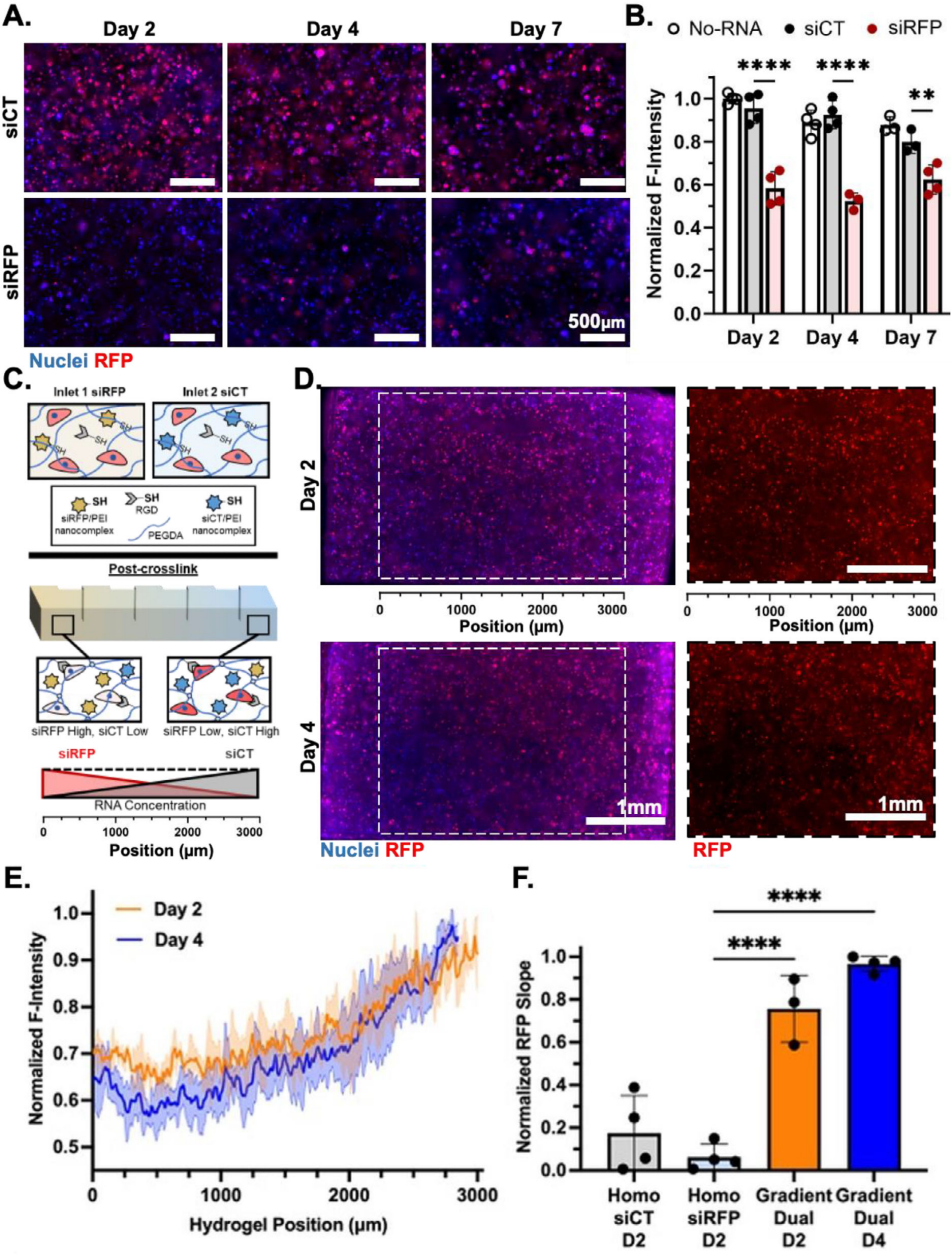
Gradient Knockdown of RFP Expression in Encapsulated Cells in Casted and Microchip Hydrogels. (A) Fluorescence microscope images and (B) quantification of RFP intensity demonstrated the ability of lyophilized siRFP/PEI‐SH nanocomplexes in silencing the RFP expression in RFP‐expressing OVCAR cells co‐encapsulated within the casted hydrogels (*n* = 4, ^**^ *p* < 0.01, ^****^ *p* < 0.0001). (C) Schematic of dual‐opposing siRNA‐gradient hydrogel generated within the fluidic device, where polymer solutions at both inlets contain the same cell density but different types of siRNAs. (D) Fluorescence photomicrographs (RFP: red; cell nuclei stained with DAPI: Blue) and (E) quantification of RFP intensity (*n* = 3–4) demonstrated that the gradient presentation of siRFP in the microfluidic chip hydrogels resulted in a corresponding gradient of RFP silencing in hydrogel encapsulated cells. White dotted lines in D denote a 3 mm region of interest. (F) Gradient slope of cell RFP intensity profile across the entire 3mm in the hydrogels encapsulated with homo‐ or gradient‐concentration RNA (*n* = 3, ^****^ *p* < 0.0001).

We sought to homogeneously encapsulate cells into the gradient siRNA‐laden hydrogels to test gene silencing via the delivery of functional siRNA in a corresponding gradient fashion. To this aim, we employed a red fluorescent protein (RFP)‐expressing OVCAR cell line and siRNA against RFP (siRFP, sense strand: GCU CCA AGG CCU ACG UGA A) to silence the expression of RFP mRNA in the cells. A non‐targeting siRNA (sense strand: GAU UAU GUC CGG UUA UGU A) was used as a control RNA (siCT). We first confirmed the ability of siRFP/PEI‐SH nanocomplexes in silencing the RFP expression in RFP‐expressing OVCAR cells in a monolayer. Compared to the absence of siRNA (No‐RNA) and non‐targeting siRNA (siCT), siRFP at both 40 and 80 nm concentrations significantly reduced RFP expression 2 days following transfection (Figure S5), indicating the transfection ability of the synthesized PEI‐SH and the bioactivity of siRFP in silencing RFP expression. To achieve knockdown in 3D hydrogels, we have previously shown that much higher concentrations, in the range of 40 µg/mL (or 3.2 µm) of siRNA, are required to visualize reduced protein expression [13, 15]. To be able to encapsulate a high concentration of siRNA into the hydrogel, lyophilized siRNA/PEI‐SH nanocomplexes were prepared with the addition of sucrose as a lyo‐protectant to preserve the bioactivity of the siRNA [10, 14, 15]. The bioactivity of the lyophilized siRFP/PEI nanocomplexes was confirmed via imaging and flow cytometry with minimal reduction compared to the fresh prepared nanocomplexes (Figures S5 and S6). Next, the lyophilized nanocomplexes (3.2 µm) were homogenously encapsulated with RFP‐expressing OVCAR cells (5 × 10^6^ cells/mL) into photocrosslinked PEG‐DA hydrogels (final PEG‐DA 10% w/v, LAP 1 mg/mL, RGD‐SH 1 mg/mL), which were cast and crosslinked at a thickness of 400 µm using the same UV intensity used when crosslinking the fluidic chip gel (110 mW/cm^2^ for 15 s), to examine the bioactivity of siRFP in 3D hydrogels. Fluorescence images demonstrated a significant reduction of RFP signal in the siRFP‐encapsulated group at all investigated time points compared to corresponding siCT and No‐RNA groups, while a similar signal of cell nuclei stained with Hoechst dye was observed (Figure 3A; Figure S7). Quantification of RFP expression indicated that the cell RFP signal in siRFP‐loaded gels was significantly silenced by ∼40% on days 2 and 4, and by ∼25% on day 7 in comparison to the No‐RNA and siCT groups (Figure 3B). This reduction in RFP signal reflects a functional knockdown of RFP expression via RNAi delivery, rather than the distribution of fluorophores as presented in previous figures. In this context, the quantified decrease in fluorescence intensity corresponds to reduced expression of the RFP protein, allowing evaluation of the silencing activity directly within the hydrogel. Prior to the delivery of gradient siRNA/PEI nanocomplexes within PEG‐DA microfluidic chip hydrogels, the cytocompatibility of the fluidic fabrication process was confirmed. NIH3T3 fibroblasts were loaded within the polymer solutions supplemented with RGD‐SH (1 mg/mL), followed by fabricating the photocrosslinked fluidic hydrogels using the same fabrication parameters as described previously (flow rate 50 µL/min, UV crosslinking at 110 mW/cm^2^ for 20s). Thiolated‐RGD cell‐adhesion peptides were covalently linked to the hydrogel using similar UV‐induced mechanisms to enhance cell attachment and survival within the PEG‐based hydrogels [13, 34]. The bulk chip hydrogels were sectioned into multiple individual sectioned hydrogels (3 × 4.5 × 3, mm), which were then cultured independently for live/dead staining and measurement of metabolic activity. Cell viability was maintained >88% and metabolic activity increased for up to 7 days following fabrication (Figure S8), demonstrating that the fluidic hydrogel fabrication process is cytocompatible for cell encapsulation and further culture.

After confirming the functionality of siRFP/PEI nanocomplexes within 3D hydrogels and the biocompatibility of the fluidic biofabrication process, we utilized our fluidic RNA gradient device to achieve a near‐linear continuous gradient gene knockdown within the cells encapsulated in the hydrogel. To do this, two PEG‐DA macromer solutions containing the same concentrations of OVCAR cells, RGD, PEG‐DA, and LAP photoinitiator (PEG‐DA 10% w/v, LAP 1 mg/mL, RGD‐SH 1 mg/mL, OVCAR 5 × 10^6^ cells/mL) were prepared, supplemented with either siRFP (Inlet 1) or siCT (Inlet 2) lyophilized nanocomplexes with PEI‐SH (RNA 40 µg/mL), and fabricated into dual‐opposing siRFP/siCT‐gradient fluidic chip photocrosslinked hydrogels (Figure 3C). The ability of gradient siRFP distribution to silence cellular RFP expression was examined using fluorescence microscopy. A schematic depicts how the gradient distribution of siRFP in the hydrogel with homogenous OVCAR cells could functionally regulate the cell RFP expression in a corresponding gradient fashion (Figure 3C). Based on our previous results with the dual gradient formation within the central 3 mm across the gradient width of the crosslinking chamber, we anticipated that the siRFP concentration would range from ∼33 µg/mL at the siRFP‐High to ∼4 µg/mL at the siRFP‐Low positions. After fabrication, the 30 mm length bulk hydrogel was removed from the fluidic device and sectioned into 10 individual hydrogels (3 × 4.5 × 3, mm), which were cultured independently and imaged at designated timepoints to examine the cell RFP distribution. The RFP distribution was visualized on days 2 and 4 following fabrication, where the entire sectioned gel was imaged, and the central 3 mm portion of the gradient width was used to quantify the RFP signal. Fluorescence microscope images demonstrated a gradient expression of cell RFP signal aligning with the expected gradient distribution of siRFP in the hydrogel, where high siRFP concentration resulted in low cell RFP expression (Figure 3D). A consistent near‐linear increase of RFP expression was observed, ranging from the siRFP‐High to siRFP‐Low regions on both experimental timepoints (Figure 3E). As controls for these trends, the cell RFP distribution was examined within similarly sized hydrogels (>3 mm) encapsulated with similar OVCAR cell density and homogeneous concentration of either siCT or siRFP. As expected, no notable changes in cell RFP distribution were observed across the width of the hydrogels. In each case, the cells in siRFP‐encapsulated hydrogels expressed less RFP signal compared to those in siCT‐loaded gels (Figure S9). Importantly, the intensity of RFP in homogenously concentrated siRFP gels (40 µg/mL) was similar to or lower than that in the gradient‐dual gels (ranges ∼33–4 µg/mL) due to the enhanced silencing ability of higher RNA concentration. The slope of RFP distribution curves within the dual‐gradient hydrogels on both days 2 and 4 was significantly higher than that of the homogenously concentrated siRFP gels, confirming the achievement of a functional siRNA gradient (Figure 3F). To demonstrate uniform cell distribution throughout the hydrogels, encapsulated cells were stained with DAPI and/or calcein to visualize live cells, in tandem with the RFP signal (Figure 3; Figures S9–S11). Hoechst‐stained images showed limitations in staining the cells in the middle of the thick constructs (Figure S10A) and could be inaccurate for visualization and quantification. We evaluated samples for gradient retention up to day 7 and observed reduced fidelity of the linear slope, likely reflecting siRNA diffusion within the hydrogel or gradual release over time. Nevertheless, significant spatial knockdown of RFP expression remained evident, with clear differences between the siRFP‐high and siRFP‐low regions (Figure S10). Calcein staining showed the presence of viable cells across the width of the sectioned hydrogels (Figure S11) while the gradient trend of RFP signal was observed, confirming that the gradient of RFP signal is a result of the cell RFP silencing caused by gradient siRFP distribution.

We demonstrated the optimization of a laser‐cut template‐generated PDMS device that can facilitate the generation of a dual‐opposing gradient siRNA encapsulated hydrogel. The use of laser‐cut molds allows for more rapid prototyping of fluidic mixer designs. With a combination of fluidic control and hydrogel photocrosslinking, we can scale down the hydrogel and gradient dimensions, allowing us to reduce the size of the engineered construct to a physiologically relevant size and be small enough for potential use in animal models of interfacial tissue injuries, including osteochondral and enthesis. In this way, RNA‐gradient hydrogels are closer in size to physiological interfaces compared to micron‐ or cm‐size constructs generated using other techniques [3]. The developed thiolated PEI not only retains its transfection capacity but also allows chemical co‐crosslinking with the hydrogel network via a hydrolysable ester linkage. The RNAi molecule immobilization via chemical tethering of RNA/PEI‐SH nanocomplexes into the hydrogel backbone minimizes burst RNA diffusion and release and allows for the engineering of 4.5 mm dual‐opposing gradient hydrogels in a high‐throughput fashion. The hydrolytic degradation of ester linkages between the RNA/PEI‐SH nanocomplexes and the hydrogel backbone allows for the release of RNA over time, followed by cell uptake for gene silencing. In this report, we produce gradient hydrogels 3 mm thick and 30 mm long, which can easily be tailored to increase the throughput or scaled to fit the application. For example, this hydrogel thickness can be changed by using thinner or thicker acrylic templates to change the channel depth to match the need. Additionally, our current optimization enabled fabrication of dual‐opposing gradients of molecules with different sizes ranging from <1 nm to hundreds of nm at an optimized flow rate of 50 µL/min. This size range covers most of the bioactive molecules, including growth factors, proteins, RNA, and DNA, and higher flow rates can also be used for additional optimization and/or increased production. In this work, we optimized our fabrication strategy to generate multiple (10 at once) dual‐opposing RNA‐gradient hydrogels (3 × 4.5 × 3 mm) at a size scale that remains challenging with other photolithography or syringe‐based gradient techniques. This approach produces gradient hydrogels at a physiologically relevant size and enables subsequent studies for pre‐clinical in vivo interfacial tissue models. For example, critical size osteochondral defects in rats, rabbits, and dog models range in thickness from ∼1.4, ∼3, or ∼4 mm, respectively [35, 36]. This platform can generate multiple hydrogels at once, which can offer advantages by serving as an alternative for therapeutic interventions, replacing the need for mosaicplasties. In this way, multiple hydrogels can be cut to shape as needed to plug damaged osteochondral regions. These gradient hydrogels can also be lyophilized, stored, and rehydrated when the application is needed without or with minimal effect on gradient RNA distribution, as the RNA‐PEI nanocomplexes are tethered to the hydrogel backbone. Although we have demonstrated the RNAi molecule functionality in only one out of two gradient channels, this approach enables the evaluation of therapeutic potential RNA gradient hydrogels, such as the delivery in opposing directions of siNoggin [12, 13, 14, 15, 22] and chondrogenesis‐enhanced RNAi molecules [23, 24, 25] to support osteochondral tissue regeneration. Identification of additional therapeutic siRNA, miRNA, and/or mRNA candidates that promote differentiation toward cell types relevant to tissue interfaces represents a key next step. We have previously demonstrated that siNoggin can be used to enhance the osteogenic differentiation of hydrogel‐encapsulated hMSCs [12, 13, 14, 15, 22]. Future efforts will focus on identifying RNA candidates that promote hydrogel encapsulated hMSC chondrogenesis, enabling the use of this dual‐opposing RNA gradient system to generate osteochondral tissue constructs at a physiologically relevant size scale. Furthermore, the release of RNA from the gradient hydrogels has the potential to influence not only encapsulated cells but also neighboring cells, leading to spatially controlled gene knockdown in the surrounding cellular environment [37]. Future studies could adapt this platform to accommodate combinations of RNAi and mRNA complexes, generating dual or multi‐molecule gradients that enable graded gene expression profiles and spatial patterns of cell differentiation characteristic of tissue interfaces.

## 3 Conclusion

In summary, we demonstrated for the first time the generation of dual‐opposing gradients of two RNA molecules within hydrogels on the scale of millimeters, which has potential in engineering complex adjacent tissues with a suitable size for animal models. This approach employed microfluidic technology to tailor the spatial presentation of RNAi molecules in the hydrogels in the form of siRNA/PEI‐SH nanocomplexes. The spatial gradient distributions of RNAi molecules were further immobilized during UV crosslinking of the hydrogels by covalently tethering the PEI‐SH to the hydrogel backbone, which helped to retain the gradient distribution for a prolonged period in millimeter scale. The whole biofabrication process is cytocompatible with minimal impact on cell viability, and the gradient‐encapsulated siRNA in the gels silenced cell gene expression in a corresponding gradient fashion. Dual opposing RNA gradients within these hydrogels can be used to guide the differentiation of hMSC, enabling the generation of interfacial tissues, such as the bone and cartilage found in osteochondral tissue, at physiologically relevant sizes that may facilitate the future application in pre‐clinical in vivo models. In addition, this approach enables the fabrication of multiple constructs per device and can be further scaled to incorporate opposing gradients with three or more components. This approach is valuable in engineering hydrogels with dual‐opposing gradients of RNAi and/or other bioactive molecules for the potential future generation of interfacial complex tissues with improved physiological relevance.

## 4 Experimental Section/Methods

### 4.1 COMSOL Model

COMSOL (v5.3) was used to model the fluidic device. The pattern of the channels was designed in Autodesk Inventor and exported as an STP file into COMSOL. Fluid dynamics were modeled using the Laminar Flow and Transport of Diluted Species modules. The device structure was composed of the channels of the fluidic device with cylinders representing the inner cannulas of 21G blunt‐tipped needles used as inlets and outlets over the appropriate locations. For the Laminar Flow simulation, the fluid was simulated as 10% PEG‐4000 in PBS with a density of 1015.3 kg/m^3^ and dynamic viscosity of 1 mPa*s, similar to PBS alone. Volumetric flow rate was varied through both 21‐G needle inlets. All surfaces that were neither an inlet or outlet were modeled as walls with the no‐slip condition. For the Transport of Diluted Species simulation, the temperature was set to 293.15 K (20°C). The diffusion coefficients for rhodamine B, PEI (MW 25 kDa), and PEI nanocomplexed with siRNA (∼250 nm) were 4.5 × 10^−10^, 0.86 × 10^−10^, and 0.017 × 10^−10^ m^2^/s, respectively. All diffusion was assumed to be isotropic. All channel walls were set to be no‐flux boundaries with initial values for concentration of all molecules at 0 mol/m^3^. The concentration of siRNA/PEI nanocomplexes was set to 1 mol/m^3^ at the inlet. The simulation was run on a physics‐controlled mesh with coarse element size.

We performed a parametric sweep of inlet volumetric flow rates, including 1, 10, 25, 50, 100, and 200 µL/min, and conducted a stationary study to investigate steady‐state conditions. The output results for fluid velocity, pressure, and concentration of PEI complexed with siRNA were collected. Visualizations were made from top‐down cross‐sections of the channels, as well as side‐view sections of the large channel, to analyze the resulting gradients created. 1D lines were drawn at a height of 1.5 mm across the large channel at 5 mm increments down the length of the channel to observe mixing and diffusion.

### 4.2 Material Synthesis

PEG‐DA was synthesized using the protocol in our previous report [14, 15, 17]. Briefly, PEG (5 mmol, MW 4 kDa, Sigma) and triethyl amine (TEA, 40 mmol, Sigma) were dissolved in 175 mL anhydrous toluene (Sigma) in a 500 mL 3‐neck round flask under stirring condition followed by immersing the flask in an ice bath. Acryloyl chloride (AC, 40 mmol, Sigma) in 25 mL toluene in a drop‐wise funnel was added dropwise to the PEG and TEA‐containing flask. 10 min after completely adding AC, the reaction flask was then removed from the ice bath and continued at room temperature under stirring conditions for 15 min, followed by continuing the reaction for 16 h at 40°C. Reaction solution was vacuum filtered to remove unwanted salt, and the collected filtrate was precipitated in ether/n‐hexane mixture solvents (2:1, v/v, both from Fisher) to obtain raw PEG‐DA powder. To further purify, the raw PEG‐DA was hydrated with deionized water (diH_2_O) and dialyzed against diH_2_O at 4°C using a dialysis membrane (3500 Da cutoff, Spectrum Laboratories Inc) for 3 days with water change twice a day. The polymer solution was then frozen and lyophilized until dry and stored at −20°C for future use. The conjugation efficiency was over 90% as confirmed by ^1^H NMR.

Thiolated cell adhesion peptides containing the amino acid sequence RGD (i.e., Cys‐Gly‐Gly‐Gly‐Arg‐Gly‐Asp‐ Ser‐Pro or CGGGRGDSP, denoted RGD‐SH) with a free thiol group in the cysteine‐ended amino acid were purchased from GenScript and coupled into the hydrogel network to improve the hydrogel attachment of the encapsulated cells.

PEI‐SH was synthesized by coupling cystamine bisacrylamide (CBA, Fisher) into the PEI (MW 25 kDa, Sigma), via the reaction of primary amine groups in PEI and acrylamide groups in CBA, followed by using dithiothreitol (DTT, Fisher) to decouple the disulfide bond in CBA. Briefly, PEI (1.0 g) and CBA (100 mg) were dissolved in 13 and 2 mL of methanol (Sigma), respectively. This formulation allows for synthesizing PEI‐SH with ∼15 thiol groups per PEI molecule. The CBA solution was dropwise added to the PEI solution at 60°C under stirring condition and the reaction was carried out for 24h, followed by vacuum removing solvent and hydrating in 15 mL diH_2_O containing 100 mg DTT. The solution was then dialyzed against diH_2_O at 4°C using a dialysis membrane (3500 Da cutoff) for 1 day with 5 water changes, followed by freezing and lyophilizing until dry and stored at −20°C for future use.

### 4.3 Cell Culture

NIH3T3 (ATCC CRL‐1658) and OVCAR‐5/RFP (Cell Biolabs AKR‐254) were cultured in DMEM High Glucose (ThermoFisher) supplemented with 10% Fetal Bovine Serum (ThermoFisher) and 100 U/mL penicillin/streptomycin (ThermoFisher). OVCAR‐5 cultures were additionally supplemented with 0.1 mm MEM Non‐Essential Amino Acids (NEAA), 2 mm L‐glutamine, and 1 µg/mL Puromycin. Cultures were maintained in an incubator at 37°C with 5% CO_2_ and relative humidity.

### 4.4 Device Fabrication

Clear cast acrylic sheets with 3.175 mm height (McMaster Carr) were laser‐cut (Universal Laser Systems) based on the design file (CorelDRAW) to generate the positive mold of the fluidic mixer. This design included circular inlets (4 mm diameter) that feed into three 0.5 mm width mixing channels (6 turns, two layers), leading to 5 channels that pool into a larger chamber at 4.5 mm width, 3 mm thick, 30 mm length. The acrylic fluidic design was immobilized onto a laser‐cut acrylic base (70 × 35 mm) using Weld On #4 (Amazon). After making sure all features are attached solidly to the base, the adhesive was allowed to cure overnight. Subsequently, Sylgard 184 polydimethylsiloxane (PDMS, Fisher) was prepared at a 10:1 elastomer:curing ratio, mixed, and desiccated prior to pouring onto the acrylic template. Then, the PDMS was desiccated again prior to curing at 80°C in an oven for at least 2 h. PDMS can be removed from the acrylic template, generating ∼3 mm negative features with the intended fluidic mixer design. 2 mm biopsy punches (Fisher) were used to puncture two inlet holes within the circular inlets, as well as one outlet at the base of the large crosslinking chamber.

### 4.5 Experimental Fluidics Optimization

For fluidics experiments, PDMS channels were either (1) irreversibly sealed with air plasma (Plasma Prep II, SPI Supplies) mediated bonding to 75 × 50 mm glass slides or (2) reversibly held together between one acrylic sheet and a Gel Slick (Fisher) treated glass slide with binder clips. Luer‐lock tubing with 2 mm outer diameter (Terumo) was used to connect dual syringe‐pump (Harvard Apparatus) loaded syringes to the PDMS device. PEG‐DA was prepared at 10% w/v in PBS supplemented with 1 mg/mL Lithium phenyl‐2,4,6‐trimethylbenzoylphosphinate (LAP) photoinitiator. Fluorescein (Sigma) and rhodamine B (Sigma) were prepared at 1 mg/mL and added separately to the PEG‐DA solution for a final concentration of 0.01 mg/mL. These fluorescent‐containing macromer solutions served as the two inlets into the fluidic device. The flow rate, set by the syringe pump, was modulated between 10 and 100 µL/min and allowed to equilibrate for at least 10 min prior to fluorescence imaging at the base, middle, and end positions with a Zeiss Axio Observer Z1. The entire 4.5 mm width of the channel was captured as two images and stitched together using ImageJ (FIJI, Pairwise stitching plugin). Fluorescence intensity of both fluorescein and rhodamine along the x‐axis was measured using ImageJ Plot Profile. For variable flow rate experiments, fluorescence intensities at each flow rate were independently normalized to the maximum and minimum signal across the three positions along the length of the crosslinking channel. For verification of a 50 µL/min flow rate, three independent fluidic devices equilibrated at 50 µL/min were measured at five positions. For each device, fluorescence intensities were first normalized to the maximum and minimum signal across all five positions to generate five curves. The fluorescence intensity curves at each position were averaged (*n* = 3) prior to comparison between positions 1 and 5 shown in Figure 1E (average ± standard deviation between all five positions).

### 4.6 Crosslinking Optimization

PEG‐DA solution was prepared at 10% w/v in PBS supplemented with 1 mg/mL LAP and either 0.01 mg/mL rhodamine B or Alexa Fluor 488 (AF488) carboxylic acid, tris(triethylammonium) salt (ThermoFisher). The fluidic devices were filled at 50 µL/min with the two inlet solutions and allowed to equilibrate for 5 min. Flow was stopped immediately prior to UV crosslinking at 110 mW/cm^2^ for 20s using an Omnicure2000 and collimating adapter (Excelitas), where uniform light was applied to the entire crosslinking chamber. Light intensity was measured using the LS200 Sensor (Excelitas). The resulting bulk hydrogel (3.0 × 4.5 × 30, mm) was removed from the PDMS device, and either imaged directly (Bulk View, coronal plane) or sectioned into 10 individual hydrogels of 3 mm in length prior to imaging through the cross‐section view (axial plane) of individual gels. This was performed using a razor blade to slice parallel to the gradient axis. In the bulk view, fluorescence intensities at each position were normalized to the maximum and minimum signal of the entire bulk hydrogel. For the cross‐section view analysis, fluorescence intensities were normalized to the minimum/maximum within each individual sectioned hydrogel due to the potential difference in thickness.

### 4.7 siRNA/PEI‐SH Nanocomplex Generation and RNA‐Gradient Hydrogel Formation

AllStars Negative Control siRNA (Qiagen) was used to visualize the distribution of siRNA throughout the hydrogel. AF488‐siRNA and AF546‐siRNA were reconstituted at 100 µm in water, and PEI‐SH solution was prepared at 2 mg/mL in PBS. siRNA/PEI‐SH nanocomplexes were prepared at an N:P ratio of 11. In separate tubes, PEI‐SH was prepared at 60 µg/mL, and siRNA was diluted to 3.2 µm (∼40 µg/mL) in PBS. siRNA solution was pipetted into the PEI‐SH solution, mixed, then vortexed briefly. Both AF488‐siRNA and AF546‐siRNA nanocomplexes were allowed to form for 10 min at room temperature. Following, these nanocomplexes were combined 1:1 (v/v) with PEG‐DA solutions (20% w/v, LAP 2 mg/mL) in PBS. These siRNA/PEI‐SH nanocomplexes contained PEG‐DA solutions served as the feeding solutions for the two inlets of the fluidic device, primed and equilibrated at 50 µL/min, then crosslinked at 110 mW/cm^2^ for 20s. The resulting bulk hydrogel (3.0 × 4.5 × 30, mm) was removed from the PDMS device, imaged directly (Bulk View) or sectioned into 10 individual hydrogels of 3 mm in length prior to imaging the cross‐section of individual gels. Normalization was performed in the same manner as previously, where the Bulk View was normalized to a “global” maximum and an empty PEG‐DA hydrogel as the negative. Individual cross sections were normalized to an independent maximum and an empty PEG‐DA hydrogel as negatives. Measurements were not included if the hydrogel contained significant edge effects, where slopes would aberrantly increase near the edges (*n* = 1 for AF488‐siRNA and *n* = 1 AF546‐siRNA). For release studies, individual cross‐sectioned hydrogels were cultured in PBS at 37°C for up to two weeks. The same gels were imaged during the course of the study to track the change in fluorescence intensity over time. The average fluorescence curve at each time point was normalized to maximum intensity at Time 0, and an empty PEG‐DA hydrogel was used as the negative. The fluorescence slopes of individual hydrogels were used to calculate changes in slope over time.

For gene silencing in 3D, siRNA was formed nanocomplexes with PEI‐SH (N/P ratio of 11 and 1 µg siRNA per total 80 µL PBS) for 25 min, followed by mixing with the same volume of sucrose in PBS (10%, w/v), freezing −80°C prior to lyophilization to obtain lyophilized nanocomplexes, as our previous report [12, 13, 14, 15]. Prior to preparing the cell and RNA encapsulated hydrogels, stock solutions of PEG‐DA and RGD‐SH (PEG 20% w/v, RGD‐SH 2 mg/mL) and LAP (25 mg/mL) in PBS were prepared. Lyophilized siRNA/PEI‐SH nanocomplexes were resuspended at 160 µg/mL with sterile water. OVCAR cells from a confluent monolayer were trypsinized, centrifuged at 300G for 5 min to get the cell pellets. Cell pellets were resuspended immediately prior to mixing at 3.3–6.6 × 10^7^ cells/mL in PBS. All solutions were combined to achieve the final pre‐hydrogel solution concentration of 10% w/v PEG‐DA, 1 mg/mL LAP, 1 mg/mL RGD‐SH, 40 µg/mL (3.2 µm) siRNA, and 0.5–1 × 10^7^ cells/mL. Small hydrogel droplets were prepared by depositing 20 µL of cell‐laden pre‐hydrogel solution onto a nylon brick (McMaster Carr) and capped with a 400 µm spacer and GelSlick‐treated glass slide. UV was applied at 110 mW/cm^2^ for 15s to crosslink and generate small hydrogel disks, which were then transferred to individual culture. Fluorescent signal of the cells in cell‐laden hydrogels was imaged at days 2, 4, and 7 following the fabrication with a fluorescence microscope. The intensity of RFP expression was quantified using ImageJ.

The biocompatibility of the biofabrication process was accessed via live/dead staining prior to evaluating the bioactivity of the siRNA in the gradient hydrogels. Similar cell‐laden macromer solutions were prepared with RNAs, and the gradient hydrogels were prepared and cultured as previously described. The sectioned hydrogels were stained for live/dead assay and performed CCK‐8 assay to access the metabolic activity. Live/dead assay was performed by incubating cell‐laden hydrogels with 2 µm calcein AM and 4 µm EthD‐1 solutions in PBS for 30–60 min, with 10‐min PBS washes prior to imaging. Metabolic assay was performed by incubating cell‐laden gels in a 10% CCK‐8 solution in DMEM for 3 h. Subsequently, 100 µL of incubation solution was removed and measured at 450 nm using a spectrophotometer.

For evaluating the bioactivity of the siRNA in the gradient hydrogels, similar cell‐laden hydrogel solutions were prepared with either siRFP/PEI‐SH or siCT/PEI‐SH nanocomplexes, and the gradient hydrogels were prepared and cultured as previously described. Here, gels were imaged at days 2 and 4 following fabrication. The RFP expression of homogenously distributed cells was analyzed with Plot Profile, and the plot was normalized to each individual hydrogel. Linear fitting was used to determine the fluorescence slope of each hydrogel. To fabricate the large‐sized homogeneous siRNA concentration hydrogel for comparison, a similar protocol as preparing the small hydrogel disk was used to crosslink 500 µL cell‐laden pre‐hydrogel solution. In addition, the samples were also incubated with a 2 µg/mL Hoechst 33342 (Abcam) solution for 45–60 min with rocking to visualize the cell nuclei.

### 4.8 Statistical Analyses

The data is depicted as means ± standard deviations with a sample size of 3 or more. Statistical analysis was performed using one‐way analysis of variance (ANOVA) followed by Tukey's multiple comparisons test using GraphPad Prism software (GraphPad Software, San Diego, California, USA).

## Funding

The authors gratefully acknowledge support from the UCLA Eli and Edythe Broad Center of Regenerative Medicine and Stem Cell Research (T.H.), and the National Institutes of Health's National Heart, Lung, and Blood Institute (T32HL069766, T.H.), National Institute of General Medical Sciences (R01GM143485, S.L.) and National Institute of Arthritis and Musculoskeletal and Skin Diseases (R01AR069564 E.A.). The contents of this publication are solely the responsibility of the authors and do not necessarily represent the official views of the National Institutes of Health.

## Conflicts of Interest

The authors declare no conflicts of interest.

## Supporting information




**Supporting File**: advs74784‐sup‐0001‐SuppMat.docx.

## Data Availability

The data that support the findings of this study are available from the corresponding author upon reasonable request.
