# Supplementary material for: Millimeter‐Scale Dual‐Opposing RNA‐Gradient Hydrogel for Interfacial Gene Silencing
Source: Adv Sci (Weinh). 2026 Apr 7;13(29):e24144. doi: 10.1002/advs.202524144 (PMC13205676; doi:10.1002/advs.202524144)
Supplement: Supplementary file 1 — Supporting File: advs74784‐sup‐0001‐SuppMat.docx. [file ADVS-13-e24144-s001.docx]

Supporting Information

Millimeter-scale Dual-Opposing RNA-Gradient Hydrogel for Interfacial Gene Silencing

Tyler Hoffman, Cong Truc Huynh*, Marcus J. Goudie, Peyton J. Tebon, Hyojin Ko, Minh Khanh Nguyen, Kaelyn L. Gasvoda, Yang Song, Kirsten Fetah, Ali Khademhosseini, Song Li, Eben Alsberg*

**
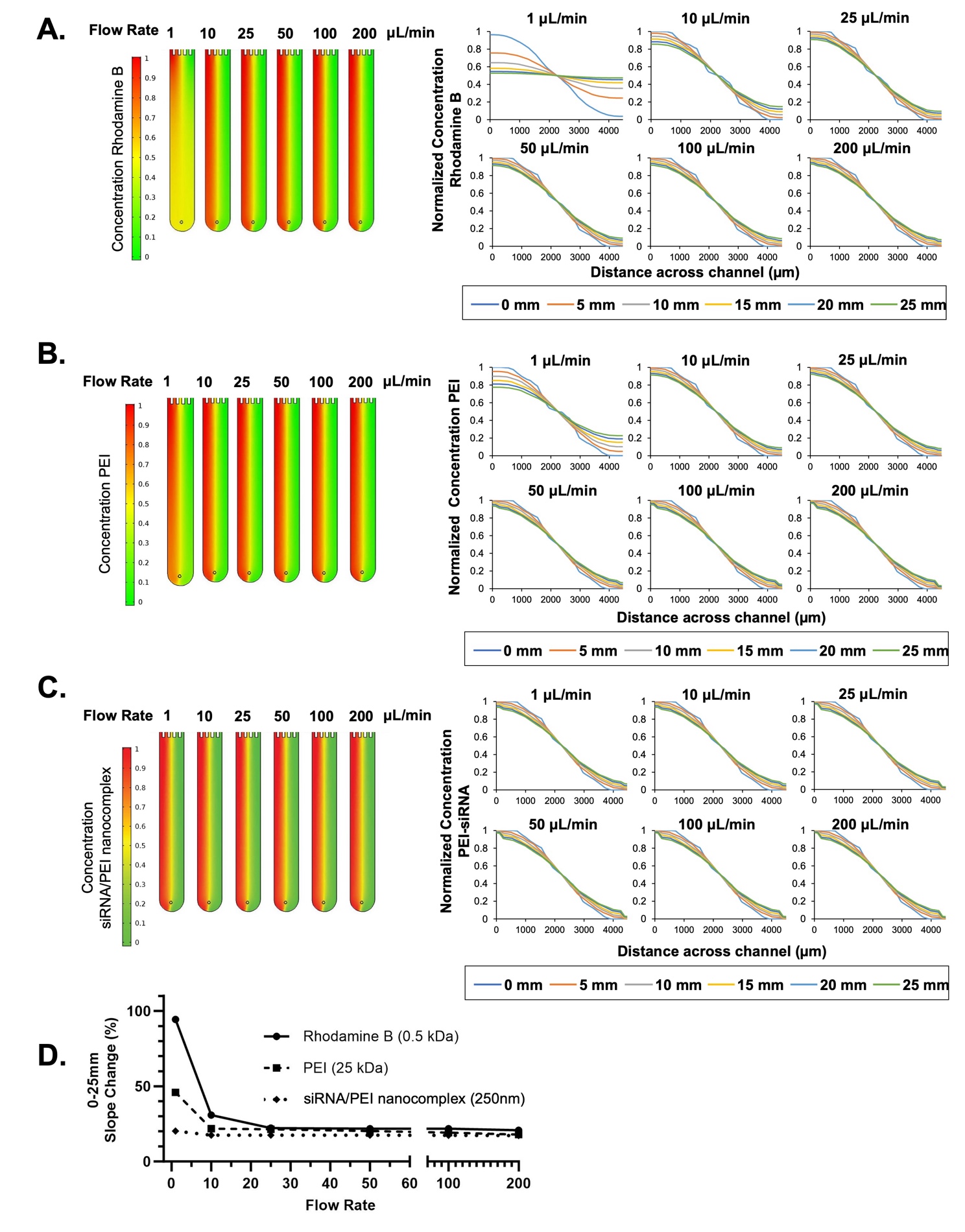
**

**Figure S1**. COMSOL modeling for the diffusion of (A) rhodamine, (B) PEI, and (C) siRNA/PEI nanocomplexes across the crosslinking chamber at varying flow rates. (D) Percent change in slope comparing the first (0 mm) to the last position (25 mm) of different molecules at varying flow rates. Each data point represents individual simulation in the COMSOL model.

**
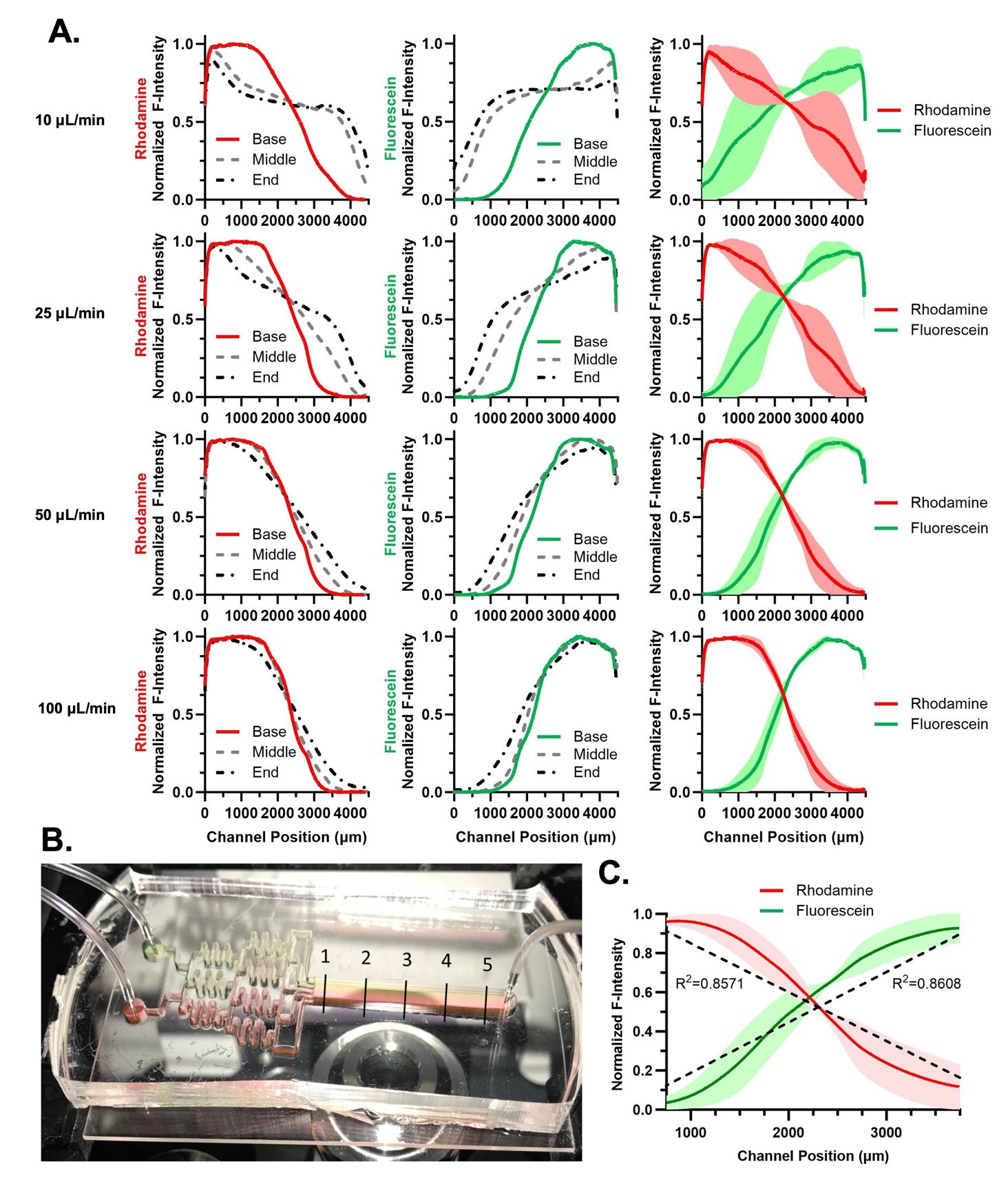
**

**Figure S2**. (A) Distribution of rhodamine B and fluorescein normalized signals across the entire gradient width of the chamber at varying flow rates within the fluidic device before crosslinking. Signals were analyzed from fluorescent images using ImageJ and normalized to the highest signal within the device. (B) Image to demonstrate the location of the imaged positions (1-5) used to quantify the fluorescent signal distribution for three individual devices at 50 μL/min to test the reproducibility of the device. (C) Line plots of rhodamine (red) and fluorescein (green) fluorescence intensities within the central 3 mm of the device. Linear fit of each profile individuated with dotted line. Plot represents average intensity with standard deviation of n=3 fluidic devices at 50 µL/min.

**
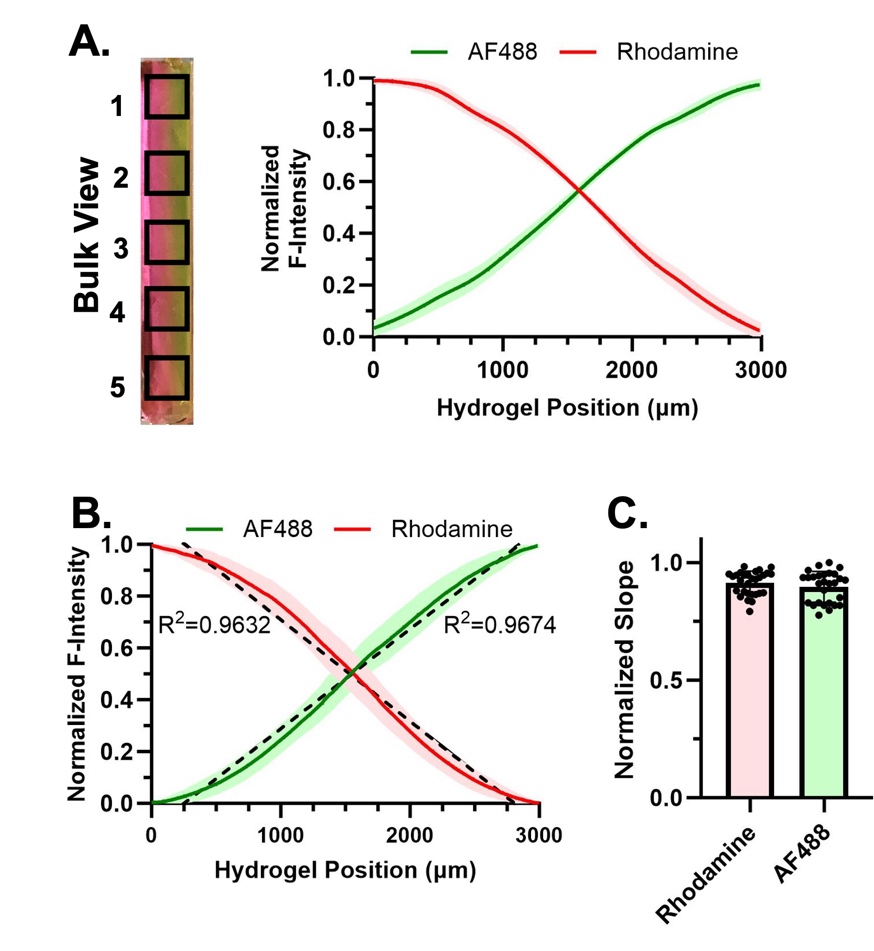
**

**Figure S3**. (A) Image of the dual-opposing gradient rhodamine/AF488 crosslinked hydrogel in the bulk view. Fluorescent images from 5 different positions were recorded, analyzed using ImageJ, and normalized to a global maximum for each fluorophore for relative intensities and distribution profiles. (B) Distribution profiles of rhodamine (red) and AF488 (green) analyzed using 30 cross-sectioned hydrogels from 3 individual chip gels. Linear fit of each profile individuated with dotted lines with no significant difference in slope. (C) Slope of each F-Intensity curve normalized to the global maximum slope.


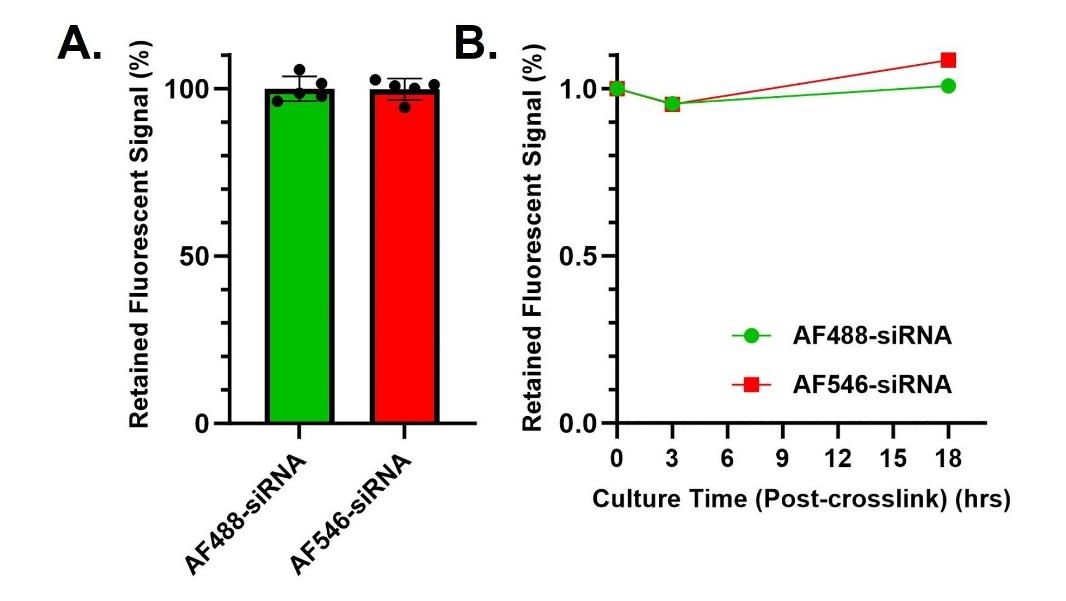


**Figure S4**. Effect of UV exposure and incubation time on the fluorescent signals of fluorescent-tagged siRNAs. Percentage of fluorescence intensity within the hydrogels: (A) after exposure to UV light (crosslinking) compared to before crosslinking, (B) after designated culture period in PBS (pH 7.4) at 37°C compared to after crosslinking.


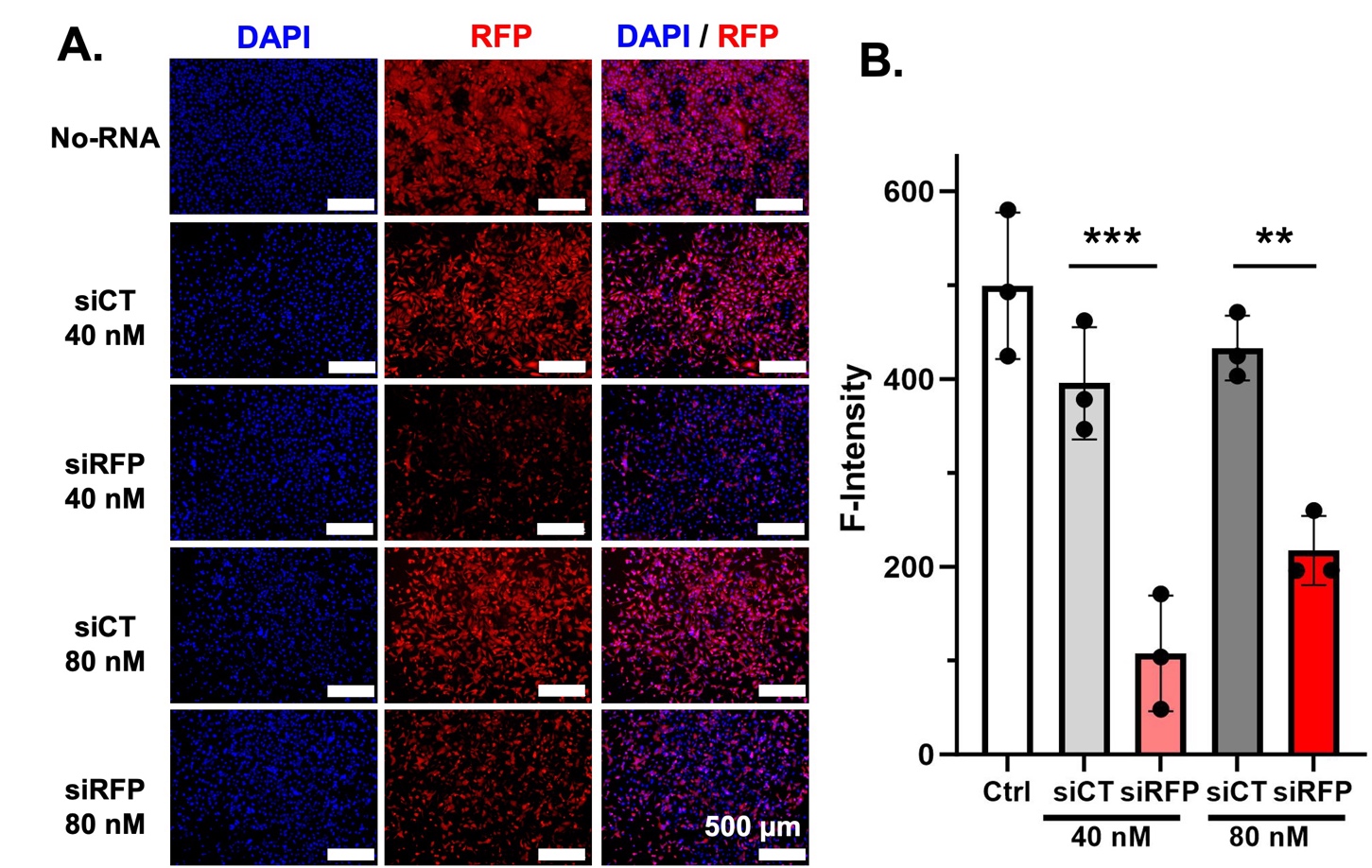


**Figure S5**. Bioactivity of fresh prepared siRNA/PEI-SH nanocomplexes. (A) Fluorescence microscope images and (B) RFP expression of RFP-expressed in OVCAR cell monolayers at 48 hours following incubation with fresh siRNA/PEI-SH nanocomplexes (n=3, ***p<0.001, **p<0.01).


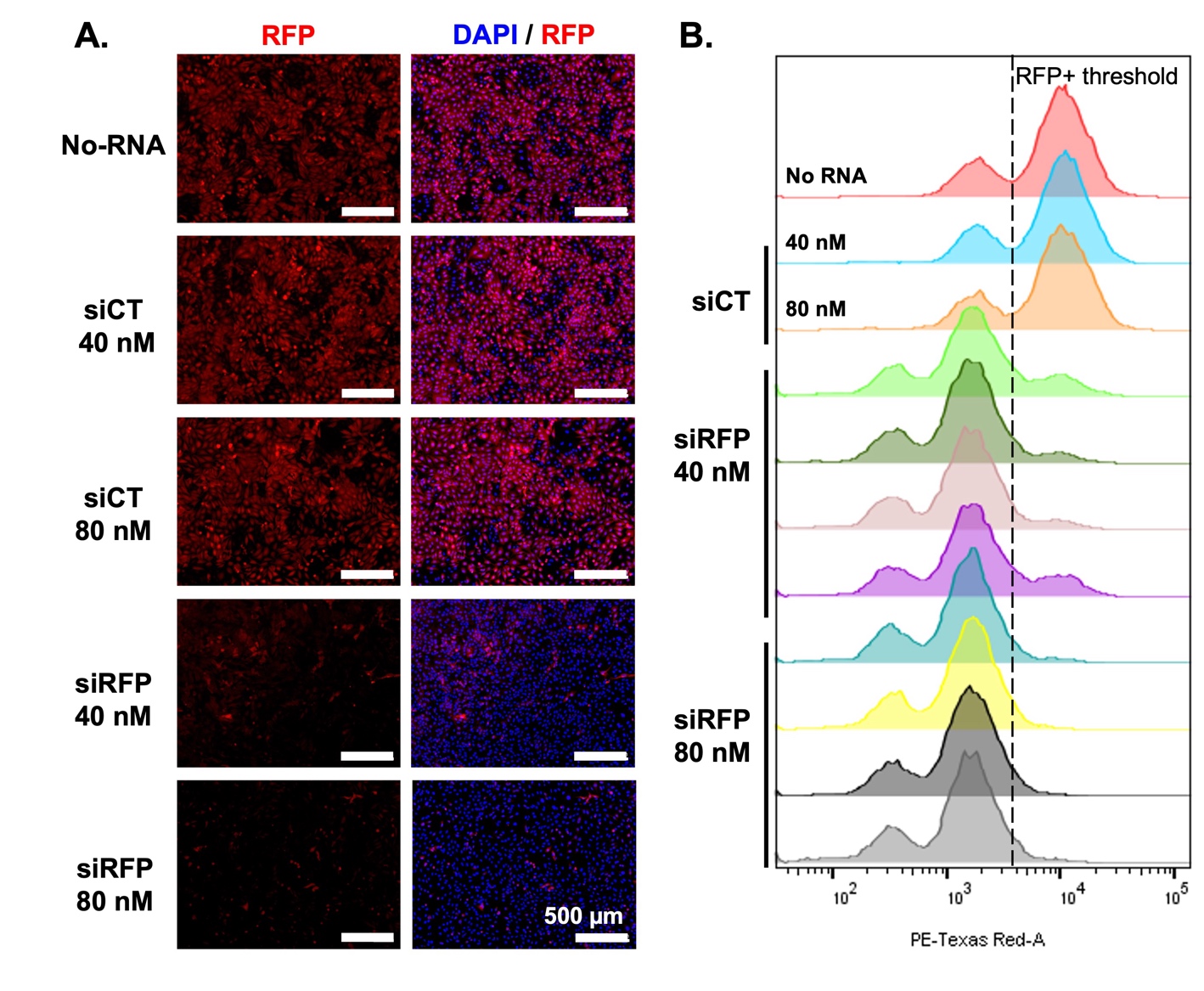


**Figure S6**. Bioactivity of lyophilized siRNA/PEI-SH nanocomplexes. (A) Fluorescence microscope images and (B) flow cytometry histogram of RFP-expressed OVCAR cell in monolayers at 48h following incubation with lyophilized siRNA/PEI-SH nanocomplexes.


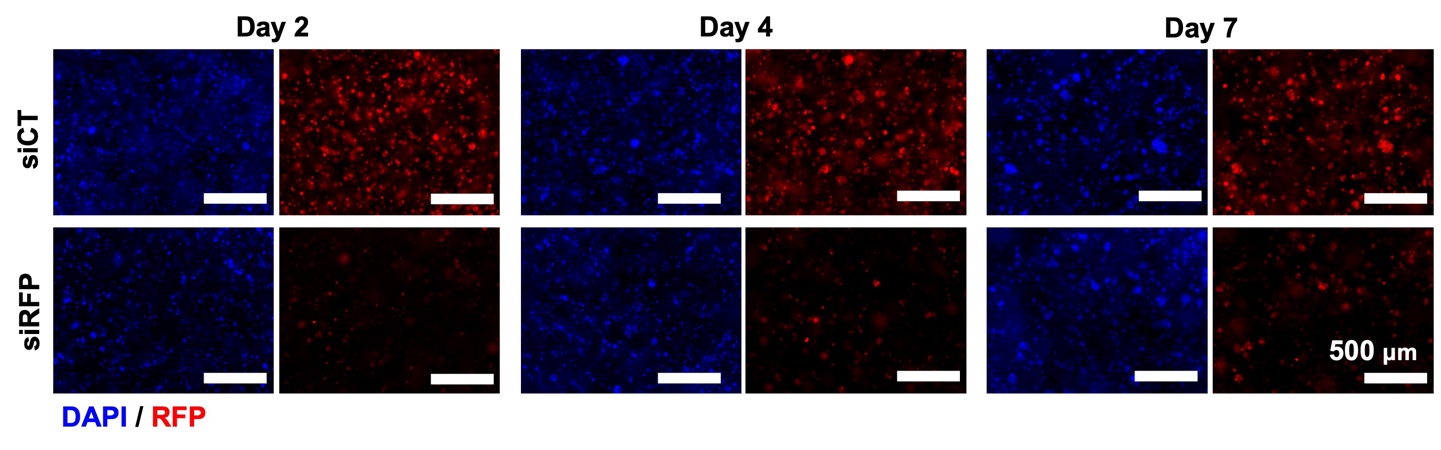


**Figure S7.** Functionality of lyophilized siRNA/PEI-SH nanocomplexes in 3D simple casted hydrogels. Single channel fluorescence microscope images of RFP-expressed OVCAR cells co-encapsulated within the casted hydrogels containing siCT or siRFP at 40 µg/mL (3.2 µM).

**
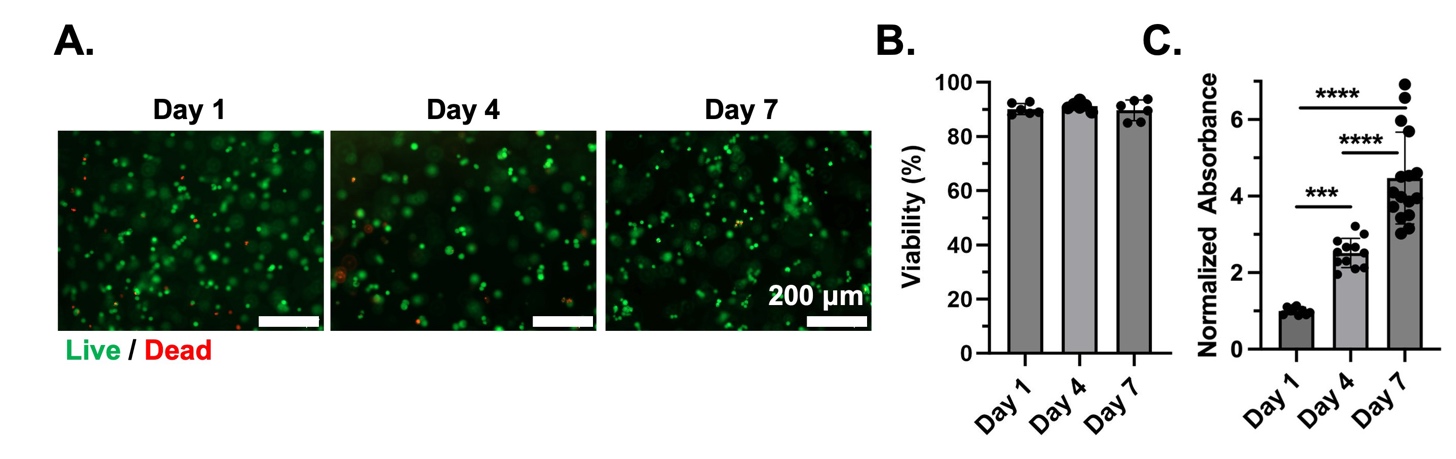
**

**Figure S8**. Viability of NIH3T3 fibroblasts following encapsulation within photocrosslinked fluidic PEG-DA hydrogels. (A) Live(green)/dead(red) staining images. (B) Fraction of green cells compared to the total number of cells (green + red) analyzed via ImageJ using live/dead image. (C) Metabolic activity measured via MTT assay and normalized to d1 (n>=9 from 3 different chips with at least 3 samples per chip, ***p<0.001, ****p<0.0001).


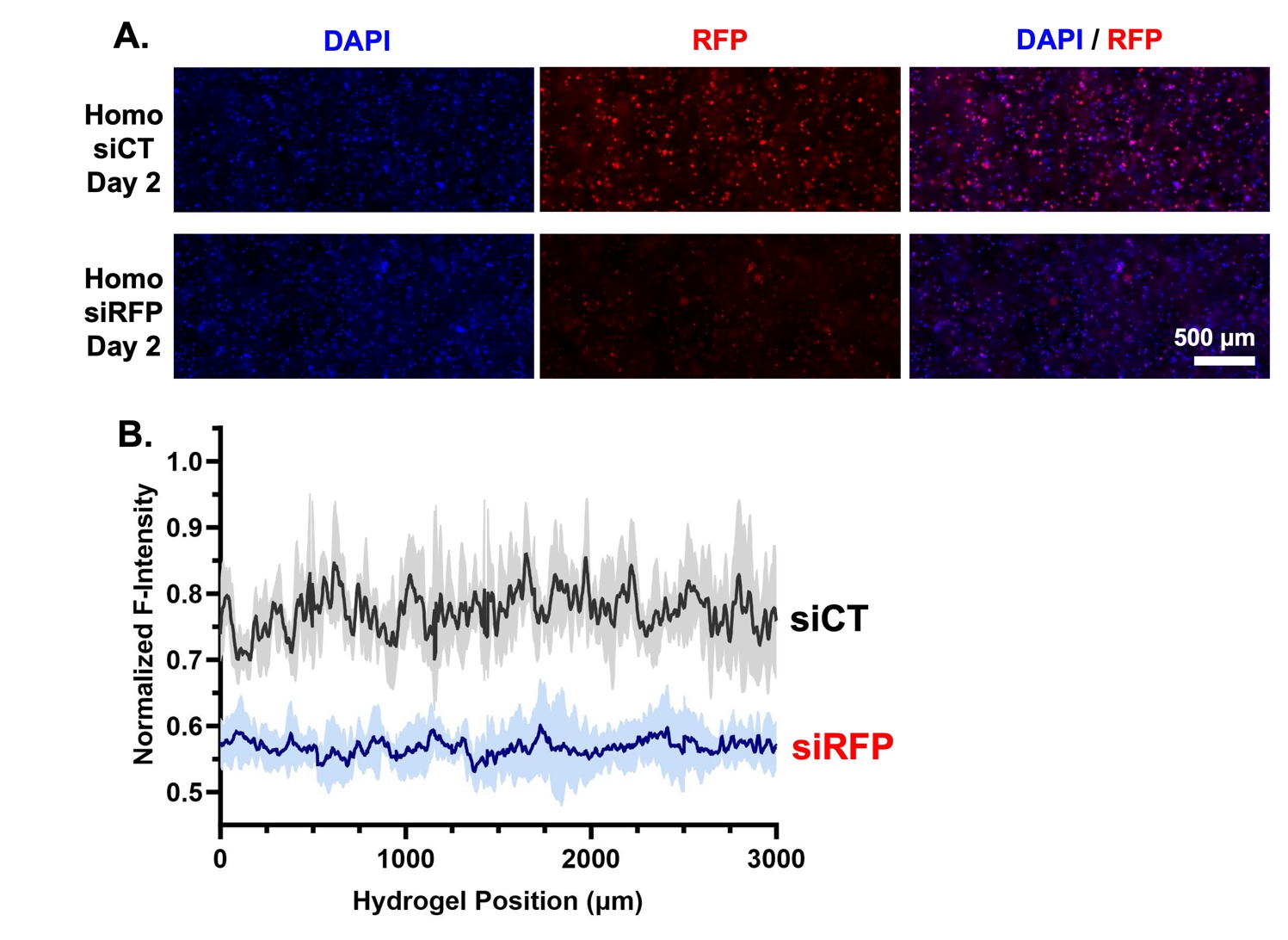


**Figure S9**. (A) Fluorescence images and (B) quantification of RFP intensity indicated the homogenous siRFP distribution in the gel could only achieve a single level of RFP expression (d2, n=4 technical replicates). Experiment was repeated 3 times with similar results.


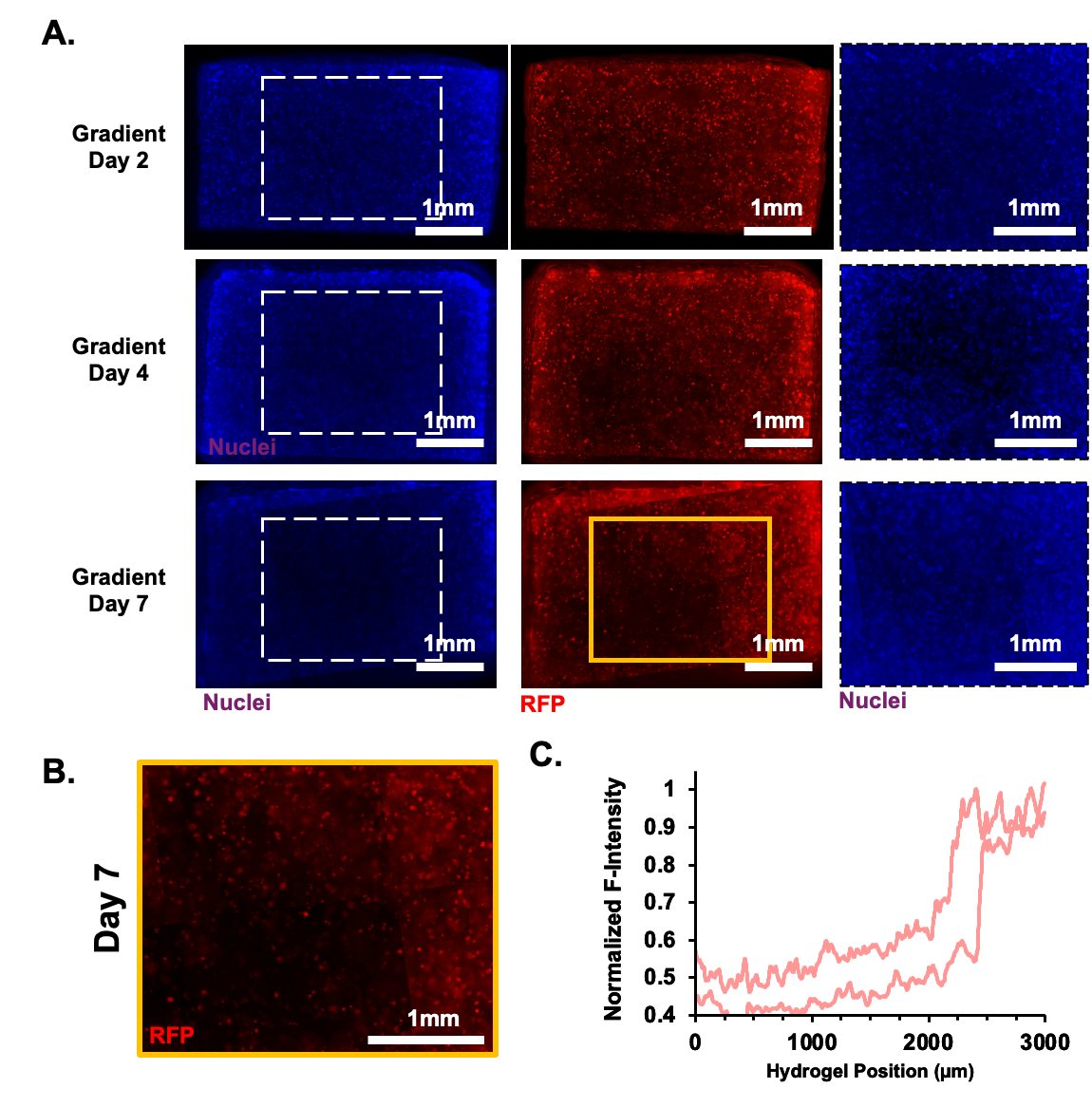


**Figure S10**. (A) Single channel fluorescence images of DAPI (blue) and RFP (red) signals of RFP-expressing OVCAR cells within dual-opposing siRFP/siCT-gradient hydrogels. (B) Single channel fluorescence images of 3-mm Region of Interest and (C) Line profiles of RFP signal at day 7 from two gradient hydrogels from (A).


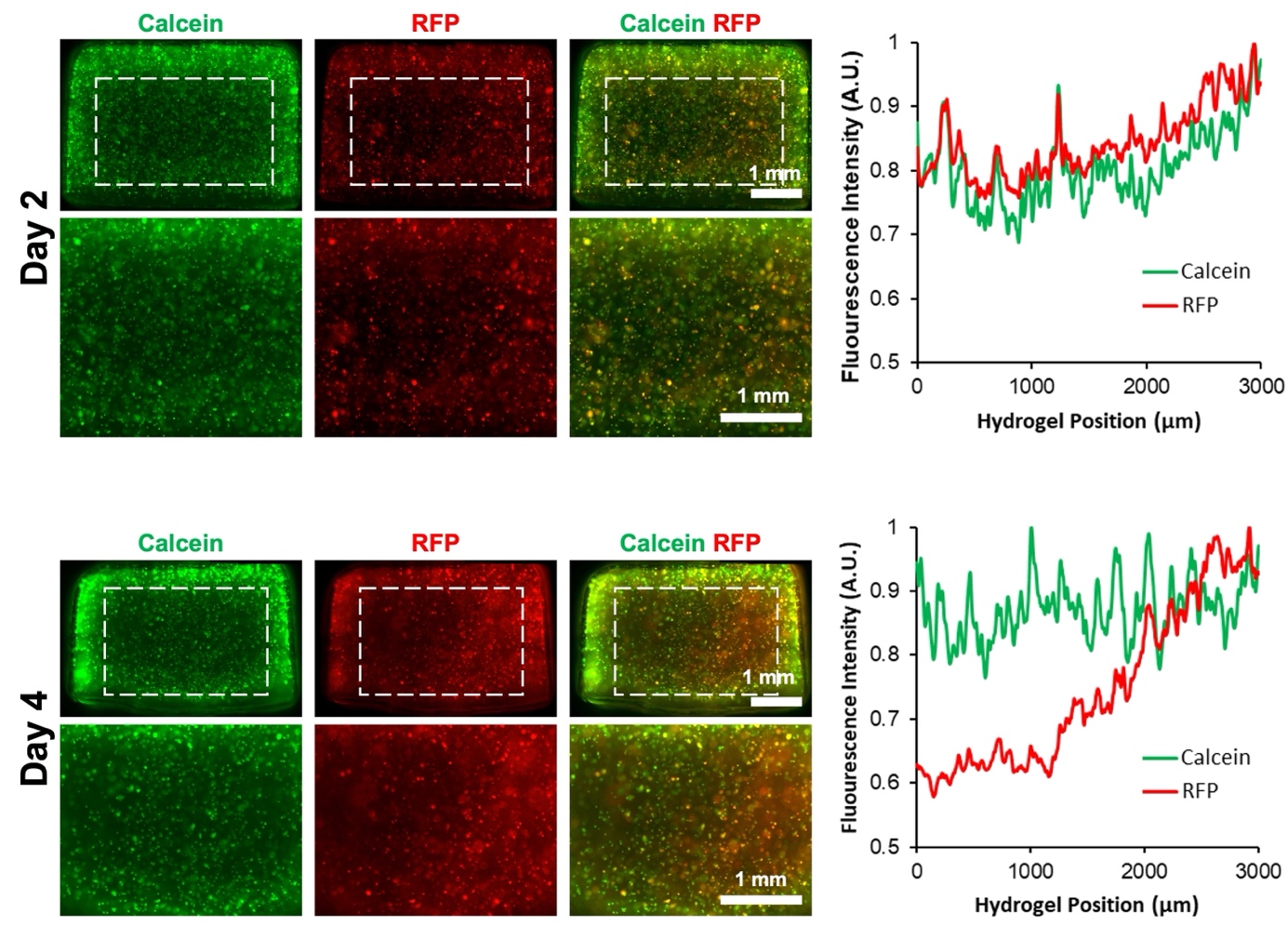


**Figure S11**. Fluorescence microscope images showing alive cell (green via calcein staining) and RFP (red) signals of RFP-expressing OVCAR cells within dual-opposing siRFP/siCT- gradients hydrogels at days 2 and 4 following fabrication. Line profiles of constitutive RFP signal (red) and Live stain (green) along the gradient width of the cross sectioned hydrogels quantified using ImageJ indicated the achievement of a gradient distribution of RFP signal with a linear alive cell signal.
